# Versatile Nanotherapeutics for Enhancing Sonodynamic Therapy/Chemotherapy of Thyroid Cancer through Remodeling Tumor Microenvironment and Synergistic Reactive Oxygen Species Augment

**DOI:** 10.34133/bmr.0338

**Published:** 2026-03-04

**Authors:** Dan Wang, Lei Sun, Juan Wang, Lirong Wang, Zhongyu Wang, Yutong Zhang, Qi Zhou, Yuhang Chen, Jue Jiang

**Affiliations:** ^1^ Department of Ultrasound, the Second Affiliated Hospital of Xi’an Jiaotong University, Xi’an, Shaanxi 710004, China.; ^2^ Department of Urology, the First Affiliated Hospital of Xi’an Jiaotong University, Xi’an, Shaanxi 710061, China.; ^3^ Department of Cardiovascular Surgery, the First Affiliated Hospital of Xi’an Jiaotong University, Xi’an, Shaanxi 710061, China.

## Abstract

Anaplastic thyroid carcinoma (ATC), as the most malignant pathological type, is prone to local invasion and even distant metastasis with a poor prognosis and a high recurrence rate. Herein, we developed an iron-based metal organic framework (FL@M) as an effective sonosensitizer and biomimetic nanocarrier through the incorporation of lenvatinib (Len) and further coating with homologous tumor cell membranes, achieving the synergistic sonodynamic therapy/chemotherapy for ATC. With the homologous tumor membrane camouflage, FL@M nanoparticles exhibited good biocompatibility, drug loading, and excellent tumor targeting ability both in vitro and in vivo. After absorption, FL@M was decomposed and released Len and Fe^3+^/Fe^2+^. Under ultrasound irradiation, FL@M exhibited excellent sonodynamic effects, rapidly generating a large amount of reactive oxygen species (ROS), which induced oxidative stress and cell apoptosis. In addition, Fe^3+^/Fe^2+^ had good catalase enzyme activity and peroxidase enzyme activity, which could catalyze H_2_O_2_ to produce O_2_ and cytotoxic •OH, respectively, further enhancing the efficacy of sonodynamic therapy (SDT). Moreover, Len exerted a synergistic effect by promoting ROS production during SDT at a lower concentration, which could decrease the occurrence of side effects. In summary, our findings demonstrated that FL@M is a safe and effective metal-organic framework-based nanoplatform to inhibit tumor proliferation, recurrence, and metastasis, offering a promising SDT/chemotherapy combination strategy on thyroid cancer.

## Introduction

Over the past 4 decades, the incidence rate of thyroid cancer has risen rapidly, ranking seventh globally and third in China among malignant tumors [1,2]. Anaplastic thyroid carcinoma (ATC) is a highly invasive malignant tumor that is prone to local infiltration and distant metastasis. Patients often have poor prognosis and high recurrence rates because of the difficulties in early diagnosis and treatment [3]. At present, surgery is the main treatment for ATC in clinical practice, supplemented by radioactive iodine (RAI) therapy and chemotherapy [4,5]. Unfortunately, there are many limitations in the clinical treatment, including recurrent laryngeal nerve injury, decreased function of the parathyroid gland, skin scars, and especially lifelong replacement medication [6,7]. Furthermore, secondary surgery faces difficulties such as tumor tissue adhesion and the inability to eradicate when patients experience local recurrence and metastasis [4,8]. Therefore, it is of great clinical significance for ATC to establish a safe and effective treatment method.

Lenvatinib (Len) is an oral multitarget tyrosine kinase inhibitor that specifically binds to vascular endothelial growth factor receptor and fibroblast growth factor receptor [9]. According to results from a clinical phase III trial, Len can effectively prolong progression-free survival and improve prognosis [10,11], which has been approved by the Food and Drug Administration for clinical treatment of differentiated thyroid cancer [11,12]. Meanwhile, many studies have reported that Len could up-regulate reactive oxygen species (ROS) levels to induce apoptosis in cancer cells and dose-dependently inhibits proliferation, migration, and invasion in ATC cells [13–16]. Nowadays, Len is currently under clinical trials for ATC treatment, but some evidences indicate unsatisfactory therapeutic outcomes because of drug resistance. Moreover, due to the poor aqueous solubility, Len exhibits low bioavailability and inadequate tumor accumulation. Effective treatment required an increase in dosage, which led to a spectrum of adverse effects including hypertension, diarrhea, fatigue, and palmar–plantar erythrodysesthesia syndrome, and others [17]. Long-term use of Len even results in drug resistance, greatly limiting its widespread application in thyroid cancer patients [11].

In recent years, sonodynamic therapy (SDT) based on low-intensity ultrasound wave has attracted widespread attention in the field of solid tumor treatment due to its advantages over traditional treatment methods, such as strong tissue penetration, good focusing, selectable irradiation sites, and minimal damage to surrounding normal tissue [18,19]. By combining sonosensitizers and noninvasive focused acoustic energy, SDT can rapidly generate a large amount of ROS, which is expected to become a new method for cancer treatment [20]. Furthermore, SDT can synergistically potentiate the sensitivity of radiotherapy and chemotherapy, thereby conferring substantial synergistic therapeutic efficacy in the management of ATC. However, due to the rapid proliferation of tumor cells and the increase of local abnormal blood vessels, the O_2_ content at the tumor site decreases, leading to a hypoxic microenvironment, thus severely limiting the effectiveness of SDT [21].

Metal-organic framework (MOF) is an emerging multifunctional nanoplatform formed by the reaction of metal ions or ion clusters with organic ligands to form a highly regular crystal framework structure [22,23]. At present, it has been applied in the biomedical field, offering important advantages such as easy synthesis and functional modification, extremely high porosity, high internal surface area and loading capacity, and good biocompatibility [24]. In particular, MOFs based on porphyrin derivatives and metal ions serve as both sonosensitizers and drug delivery platforms, which effectively solve the problems of low bioavailability and poor tumor site aggregation of sonosensitizers [25,26]. As a therapeutic nanoparticle, MOFs served several advantages over systemic administration of drugs, such as minimizing side effects, overcoming dosage limitations, and averting drug resistance [27,28], demonstrating high pharmacological activity [29,30]. Researchers have found that excessive metals, such as Ag, Pt, and Fe, can decompose endogenous H_2_O_2_ to produce O_2_ [31–33], which could effectively relieve hypoxia and enhance the killing effect of SDT on tumor cells. Furthermore, Fe can also exhibit peroxidase (POD)-like activity in catalyzing H₂O₂ into •OH [26]. Accordingly, it provides a new perspective for alleviating the hypoxic environment of tumors and offers a promising design strategy for oxygen self-loading platforms. Moreover, the biomimetic functionalization strategy based on cell membrane coating has recently sparked considerable interest in the development of intelligent nanoparticles [34,35]. It is reported that nano-delivery systems modified with homologous tumor cell membranes exhibit targeting and biocompatibility [36].

**Fig. 1.**
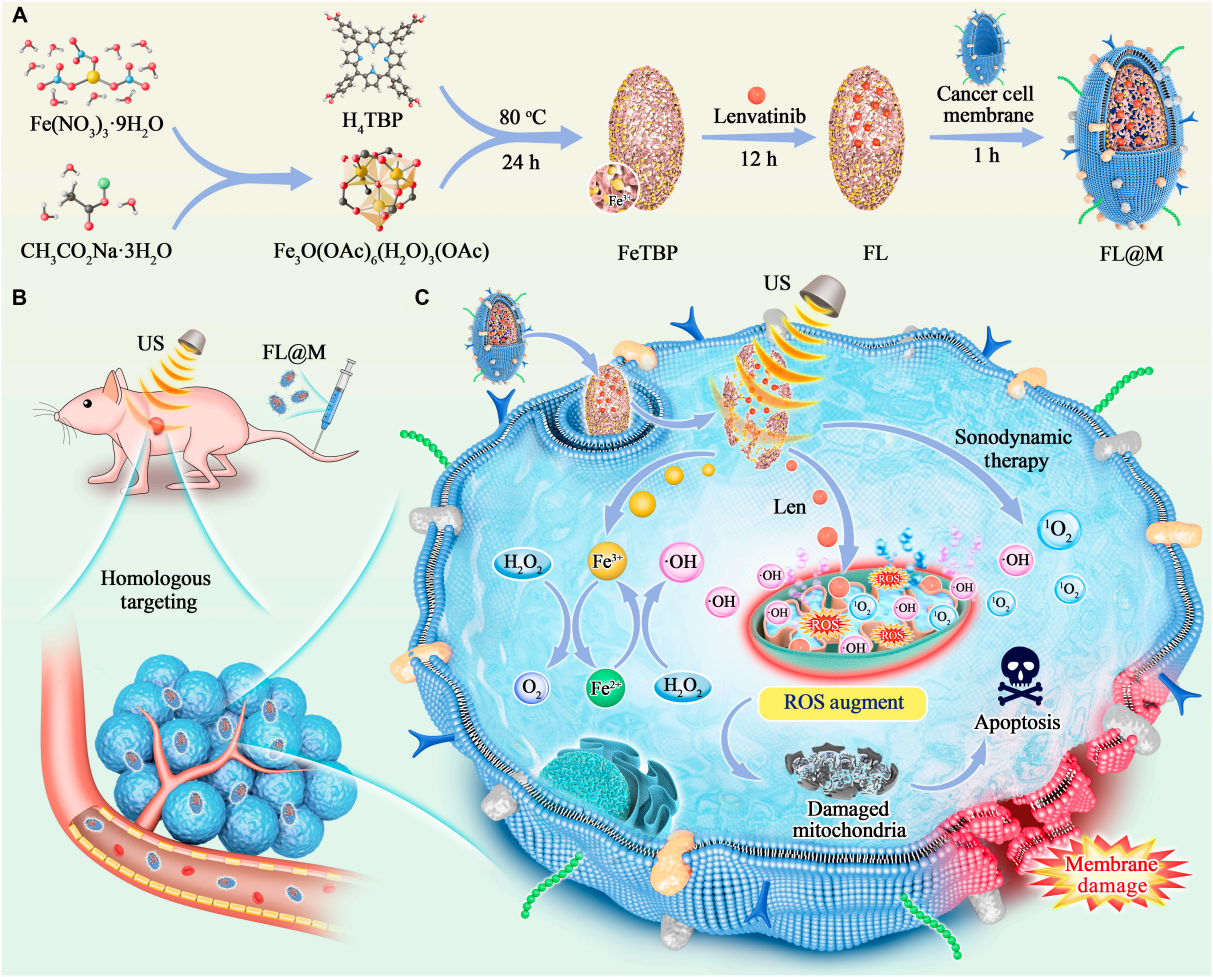
Schematic illustration of biomimetic nanoplatform FL@M for remodeling tumor microenvironment and synergistic ROS-augmented combination therapy on thyroid cancer. (A) The synthetic route of FL@M. (B) Targeted delivery of FL@M to the tumor by intravenous injection. (C) FL@M was internalized into cells through homologous targeting. After absorption, FL@M was decomposed and Fe^3+^/Fe^2+^ and Len were released. Under US stimulation, FL@M was functioned to enhance synergistic SDT/chemotherapy for thyroid tumors.

In this study, a MOF (FeTBP) based on porphyrin and Fe coated with homologous tumor cell membrane was designed to incorporate Len (FL@M) and achieve combined SDT/chemotherapy for thyroid cancer (Fig. 1). The FL@M nanocarriers exhibited an outstanding tumor cell-targeting ability through the homing effect of homologous cell membrane and then were decomposed to release Fe^3+^/Fe^2+^ and Len. Under ultrasound (US) irradiation. the sonosensitizer porphyrin was activated to produce a large amount of ROS, directly causing oxidative stress and apoptosis in cells. Besides, Fe^3+^ acted as catalase (CAT)-like actively to decompose H_2_O_2_ into O_2_ in situ, alleviating the tumor hypoxic microenvironment. Fe^2+^ could produce cytotoxic •OH by Fenton reaction and aggravate intracellular ROS production. Additionally, Len could increase ROS production and further induce cell apoptosis to enhance the treatment of thyroid cancer while reducing the systemic toxicity and side effects through targeted delivery. In all, the FL@M nanocarriers demonstrated an excellent synergistic antitumor effect, providing a novel therapeutic strategy to realize SDT/chemotherapy for thyroid tumors.

## Materials and Methods

### Materials

Fe(NO_3_)_3_·9H_2_O, CH_3_CO_2_Na·3H_2_O, 2,2,6,6-tetramethylpiperidine (TEMP), 5,5-dimethyl-1-pyrroline-N-oxide (DMPO), and Len were purchased from Aladdin (Shanghai, China). DAPI, Hoechst 33342, phalloidin-AF488, Mito-Tracker Green, JC-1 staining, Membrane Protein Extraction Kit, and 2,7-dichlorodihydrofluorescein diacetate (DCFH-DA) were purchased from Beyotime (Shanghai, China). 1,3-Diphenyl isobenzofuran (DPBF) and [Ru(dpp)_3_]Cl_2_ (RDPP) were purchased from Macklin (Shanghai, China). Calcein-AM/PI Detection Kit and Annexin V-PE/7AAD Apoptosis Detection Kit were purchased from Servicebio (Wuhan, China). Lenvatinib conjugated with fluorescein isothiocyanate (Len-FITC) was purchased from Resenbio (Xi’an, China). ROS Green probe and aminophenyl fluorescein (APF) probe were purchased from Maokang (Shanghai, China). Phenylmethylsulfonyl fluoride (PMSF) was purchased from TargetMol (Shanghai, China). Transwell chamber was purchased from BIOFIL (Guangzhou, China).

WED-100 ultrasonic treatment apparatus (Welld, China) was used as the ultrasonic trigger source (pulsed wave irradiation, frequency: 1.0 MHz, duty cycle: 50%, sonicator size: 2.0 cm^2^, pulsed repetition frequency: 10 ms).

### Cell lines and culture

The human thyroid cancer cell line 8505C was purchased from the American Type Culture Collection (USA). HeLa was purchased from the cell bank of the Shanghai Institute of Cell Biology (Shanghai, China). Both cell lines were cultured in Dulbecco’s Modified Eagle Medium (DMEM) (Hyclone, South Logan, UT, USA) with 10% fetal bovine serum (Viva Cell, Shanghai, China) and 1% streptomycin–penicillin solution (Gibco, USA). The cells were kept in a humidified incubator (Thermo Fisher Scientific, MA, USA) at 5% CO_2_ and 37 °C and passaged every 2 days with a complete culture medium change.

### Synthesis procedure

FeTBP was synthesized in 2 steps. Firstly, Fe(NO₃)₃·9H₂O (10 mmol, 4.04 g) and CH₃CO₂Na·3H₂O (20 mmol, 2.72 g) were ground into a paste in a mortar and dissolved in methanol (30 ml). Then, the reaction mixture was transferred to a round bottom flask to stir under refluxing condition for 12 h. After stirring, the reaction mixture was filtered and the filtrate was kept undisturbed for crystallization, affording dark brown crystals after several days [26]. Next, Fe_3_O(OAc)_6_(H_2_O)_3_(OAc) solution (4 ml, 2.2 mg/ml in N,N-dimethylformamide [DMF]), H_4_TBP solution (4 ml, 1.32 mg/ml in DMF), and 800 μl of formic acid were added into a 20-ml glass vial followed by keeping in an 80 °C oven for 24 h. Afterward, the reaction mixture was purified through centrifugation (12,000 rpm, 10 min) and washed with DMF and ethanol. The precipitate was then collected and vacuum-dried until a constant weight is reached, affording FeTBP.

For the synthesis of FL, FeTBP (40 μl, 50 mg/ml in DMF) and Len (20 μl, 50 mg/ml in dimethyl sulfoxide [DMSO]) were dispersed in 2 ml of ultrapure water, followed by stirring for 12 h under dark conditions. Then, the mixed solution was washed with ultrapure water (12,000 rpm, 10 min) to remove excess Len, and FL was obtained.

### Synthesis of FL@M

The Membrane Protein Extraction Kit was adopted to extract cell membranes from 8505C cells. Briefly, 1 ml of membrane protein extraction reagent A containing 10 μl of PMSF was added to the collected 8505C cells and incubated on ice for 10 to 15 min. The mixture was centrifuged (700 *g*, 10 min, 4 °C) after ultrasonic decomposition. The supernatant was then centrifuged (14,000 *g*, 30 min, 4 °C), and the precipitate was washed 3 times with ultrapure water. Then, cell membrane fragment was obtained, resuspended in ultrapure water, and kept at −80 °C. The concentration of cell membrane was detected by Nanodrop (Thermo Fisher Scientific, USA). The cell membrane fragment (1 mg) and FL (2 mg) were mixed and stirred on ice for 1 h before passing through the Avanti mini-extruder (Merck, Germany). Finally, FL@M was obtained by centrifugation (12,000 *g*, 10 min, 4 °C) to remove the excess cell membrane fragment. Similarly, F@M nanoparticles were prepared with the same procedure.

### Membrane protein characterization

The membrane proteins of FL@M were represented by sodium dodecyl sulfate–polyacrylamide gel electrophoresis (SDS-PAGE). The 8505C cell lysate and 8505C cell membrane were employed as controls. All samples (identical protein amounts) were heated at 100 °C for 10 min with a loading buffer before being separated by a 10% SDS-PAGE at 120 V for 2 h. After Coomassie blue staining and destaining in 10% acetic acid/5% ethanol overnight, the gels were imaged to observe protein distribution. Western blotting analysis determined the presence of membrane-specific protein markers (Galectin-3 and E-cadherin).

### Characterization

The chemical structure, elemental distribution, and phase chemical composition of FeTBP and FL@M were analyzed using x-ray photoelectron spectroscopy (XPS), scanning electron microscopy with energy dispersive spectrometer, and transmission electron microscopy (TEM). The particle size and potential of nanoparticles were analyzed using a Malvern particle size analyzer. The content of Fe element in nanoparticles was analyzed by inductively coupled plasma mass spectrometry (ICP-MS).

### Detection of ^1^O_2_, •OH, and O_2_^•−^ in ESR

We selected TEMP and DMPO as trapping agents to identify singlet oxygen (^1^O₂), hydroxyl radicals (•OH), and superoxide anions (O_2_^•−^), respectively. Briefly, TEMP was added to different group solutions (TEMP, TEMP + FL@M, TEMP + FL@M + H_2_O_2_, TEMP + FL@M + US, and TEMP + FL@M + H_2_O_2_ + US) with or without US irradiation (1.0 W/cm^2^) for 3 min. Then, the mixture was detected by an ESR spectrometer immediately. Similarly, DMPO was added to different concentrations of FL@M solution and detected by an ESR spectrometer. FL@M free solutions and the addition of •OOH were used as a negative control group and positive control group, respectively.

### The drug loading of FL

We determined the drug loading of FL by mixed FeTBP (2 mg) with different concentrations of Len (0.1, 0.25, 0.5, 0.75, 1.0, and 1.5 mg·ml^−1^, dissolved in DMSO). Briefly, the sample was washed with ultrapure water after stirring for 12 h, and the supernatant was collected. The concentration of Len was quantified by ultraviolet–visible (UV−vis) spectrophotometry at 242 nm. The encapsulation efficiency of Len in samples was calculated: (Input quantity of Len − Quantity of Len in the supernatant)/Input quantity of Len × 100%.

### ROS detection

Intracellular ROS level was detected using the DCFH-DA probe. In short, FL@M was mixed with DCFH-DA (2 ml, 10 μM, dissolved in phosphate-buffered saline [PBS]) followed by US irradiation for 10 min. At the predetermined time point, 400 μl of the mixture was taken out to record the fluorescence emission spectrum of DCF in the range of 500 to 600 nm, with an excitation wavelength of 488 nm. The H_2_O_2_-only (200 μM) group was set as control.

The ^1^O_2_ generation of FL@M under US irradiation was measured by the DPBF probe. In brief, FL@M was mixed with DPBF (2 ml, 100 μM, dissolved in PBS) followed by US irradiation for 10 min. Then, 400 μl of the mixture was taken out to record the absorbance of DPBF at 415 nm.

The •OH generation was measured by APF probe. In brief, FL@M was mixed with APF (2 ml, 5 μM) followed by US irradiation for 10 min. Then 400 μl of the mixture was taken out to record the fluorescence intensity at 515 nm with an excitation wavelength of 488 nm.

### Stability assessment

To detect the liquid stability of nanoparticles, the aqueous solution of FL was diluted in saline solutions with different pH values away from light. The size and zeta potential of FL were monitored for 7 days.

### Cellular uptake

The cellular uptake of FL@M was evaluated in 8505C cells. Firstly, 8505C cells were seeded on glass-bottom dishes and cultured overnight. FL or FL@M was added to cells and incubated for different times (1, 2, 4, and 7 h). After fixation with 4% paraformaldehyde, the samples were stained with phalloidin-AF488 (30 min) and DAPI (10 min) followed by imaging using a Zeiss microscopy imaging system. In order to conduct intracellular distribution studies, 8505C cells were seeded on glass dishes and treated with FL@M. Then, the cells were stained with Lyso Tracker Green DND-26/Mito Tracker Green for 30 min. After incubation, cells were washed and fixed. Then, cells were stained with DAPI (10 min) before imaging. The fluorescence intensity and colocalization was quantified by Image J software.

To further quantitatively analyze the cellular uptake capacity, 8505C cells and HeLa cells were seeded in 6-well plates and treated with FL or FL@M for 4 h. The solvent group was used as the negative control. Finally, the cells were collected and analyzed by flow cytometry (BD Biosciences, USA).

### Live–dead cell staining

The killing effect of FL@M was detected by Calcein-AM/PI staining. Briefly, 8505C cells were seeded in a 6-well plate overnight and incubated with FeTBP, F@M, or FL@M for 4 h followed by US irradiated (1.0 W/cm^2^, 3 min). Then, the samples were stained with Calcein-AM/PI and imaged by fluorescence microscopy.

### Colony formation assay

Cell proliferation capacity was monitored using colony formation assays. 8505C cells were seeded into 6-well plates (500 cells/well). The cells were treated with various nanoparticles, and the medium was refreshed every 3 days until colonies appeared. Then, the colonies were fixed by 4% polyformaldehyde for 10 min, followed by room temperature staining with 1% crystal violet for another 10 min. Colony growth was observed by counting the number of colonies. Three samples were performed every group.

### Transwell migration assay

The migratory ability of 8505C cells was assessed using a 24-well transwell chamber assay with 8-μm pore membranes. The lower chamber contained DMEM with 10% FBS as a chemoattractant, while the upper chamber was seeded with 5 × 10^4^ cells in serum-free DMEM. After 24 h of migration under standard culture conditions, nonmigratory cells on the upper membrane surface were removed with a cotton swab. Cells that migrated to the lower side were fixed with 4% paraformaldehyde, stained with crystal violet, and counted in 5 randomly selected fields per membrane under a 100× microscope.

### In vitro synergistic therapeutic effect

The synergistic therapeutic effect of FL@M was evaluated by 3-(4,5-dimethylthiazol-2-yl)-2,5-diphenyltetrazolium bromide (MTT) assay. Firstly, 8505C cells were seeded in a 6-well plate overnight and respectively incubated with FeTBP, Len, F@M, and FL@M for 4 h and followed with or without US irradiation (1.0 W/cm^2^, 3 min). After incubation for 20 h, the samples underwent MTT testing.

In addition, MTT assay was also used to detect the biological safety of FeTBP. Briefly, the 8505C cells and HeLa cells were cultured in a 96-well plate overnight. The gradient concentrations of FL@M (0, 10, 25, 50, 100, 150, 200, and 300 μg·ml^−1^) were respectively added and further incubated for 24 h before performing MTT testing. Each group set 6 parallel samples.

### In vitro ROS and lipid peroxidation detection

DCFH-DA was selected to identify total ROS in vitro. In short, 8505C cells were seeded on a 6-well plate overnight and incubated separately with FeTBP, Len, F@M, and FL@M for 4 h and followed with or without US irradiation (1.0 W/cm^2^, 3 min). Then, the cells were incubated with DCFH-DA (10 μM) for 30 min and collected to photograph using a fluorescence microscope (Leica DMC6200, Germany). In order to quantitatively analyze the ROS production, 8505C cells were treated similarly and analyzed by flow cytometry.

After being treated with various formulations for 24 h, intracellular lipid peroxide was tested with a C11-BODIPY581/591 probe (10 μM) in 8505C cells. The cells were inspected utilizing a fluorescent microscope (Leica LAS X, Germany) and flow cytometry.

### In vitro apoptosis and mitochondrial membrane potential detection

8505C cells were seeded on 6-well plates overnight and incubated with FeTBP, Len, F@M, and FL@M for 4 h followed by US irradiation (1.0 W/cm^2^, 3 min) or not. After incubation for another 20 h, the cells were collected and stained by Annexin V-PE/7AAD and then detected by flow cytometry.

Mitochondrial membrane potential (MMP) was measured using JC-1. 8505C cells were incubated with FeTBP, Len, F@M, and FL@M for 4 h followed by US irradiation (1.0 W/cm^2^, 3 min) or not. After incubation for another 3 h, cells were harvested and incubated with JC-1 at 37 °C for 30 min. Then, the samples were collected and analyzed by microscopy and flow cytometry.

### In vitro O_2_ production

Intracellular O_2_ production was detected by [Ru(dpp)_3_]Cl_2_ (RDPP) probe. Firstly, 8505C cells were seeded in a 6-well plate and cultured overnight. Then, FL@M was added to cells and incubated for 24 h before incubated with RDPP for 30 min. To mimic the tumor microenvironment, cells were transferred in an anaerobic chamber (1% O_2_) for 6 h before incubation with FL@M for another 18 h. Finally, the cells were collected and analyzed by flow cytometry.

### H_2_O_2_ decomposition assessment

Intracellular H₂O₂ decomposition was detected using the ROS Green probe. 8505C cells were seeded in 6-well plates and cultured overnight, followed by incubation with FL@M for 4 h. Thirty minutes before the end of incubation, 200 μM H₂O₂ (final concentration) was added. Next, cells were washed and 10 μM probe was incubated for another 30 min. Cells were collected and analyzed by flow cytometry. To mimic the tumor microenvironment, cells were precultured in an anaerobic chamber for 24 h prior to FL@M incubation, with all other procedures performed as described above under hypoxic conditions.

### Western blotting

The Western blotting was performed according to the method described by Wang et al [37]. Briefly, cell pellets were lysed using radio immunoprecipitation assay (RIPA) lysis buffer and the protein concentration was determined using bicinchoninic acid (BCA) protein assay (Thermo Fisher Scientific, USA). We used 15 to 20 μg of protein for Western blotting. The primary antibodies used in this study were as follows: GPX4 (ab125066, Abcam), MMP2 (10373-2-AP, Proteintech), MMP9 (10375-2-AP, Proteintech), MMP1 (10371-2-AP, Proteintech), E-cadherin (20874-1-AP, Proteintech), HIF-1α (20960-1-AP, Proteintech), Galectin-3 (82024-1-RR, Proteintech), Vinculin (A2752, ABclonal), and ACTB (AC026, ABclonal).

### Hemolysis assay

The biocompatibility of FL@M was evaluated through hemolysis assay. Firstly, fresh blood (1 ml) was washed repeatedly with PBS (3,000 rpm, 10 min) and resuspended to obtain red blood cell (RBC) suspension. Subsequently, 0.2 ml of RBC solution was mixed with 0.8 ml of FL@M solution in PBS to obtain FL@M solutions with different final concentrations (0.1, 0.2, 0.4, 0.6, 0.8, and 1.0 mg·ml^−1^), followed by incubation at 37 °C for 8 and 24 h, respectively. Then, the mixture was centrifuged (3,500 rpm, 5 min) and a microplate reader was used to detect the supernatant at 540 nm. PBS and deionized water were used as negative and positive control groups, respectively. Three parallel samples were set for each group.

$$\text{Hemolysis rate}\;\left(\%\right)\;=\frac{\mathrm{OD}\;\text{experimental group - }\mathrm{OD}\;\text{negative control}}{\mathrm{OD}\;\text{positive control - }\mathrm{OD}\;\text{negative control}}\times 100\%$$ (1)

### Animal model

All animal experiments were performed following the Principles of Laboratory Animal Care and Guidelines of the Laboratory Animal Care Committee of Xi’an Jiaotong University (No. 2021-213). All animals were purchased from GemPharmatech (Jiangsu, China) and acclimatized to the animal facility for 1 week before experimentation and housed under normal conditions with 12 h light and dark cycles and allowed free access to water and food. Briefly, 8505C cells at a concentration of 8 × 10^6^ were subcutaneously injected into male nude mice at 5 weeks old to establish a subcutaneous tumor model. It took about 4 weeks for tumors to grow approximately 70 to 100 mm^3^ before experiments.

### In vivo safety evaluation

Male KM mice (5 weeks old) were divided into 4 groups randomly (*n* = 3 per group): (a) long-term experimental group, (b) long-term control group, (c) short-term experimental group, and (d) short-term control group. Two experimental groups were intravenously injected with FL@M (5 mg/kg) while the control group was injected with the same volume of saline. Fresh blood and major organs were collected in 14 and 30 days after injection and were analyzed or sectioned for HE staining, respectively.

### In vivo fluorescence imaging, biodistribution and pharmacokinetic evaluation

Briefly, FL@M or FL-FITC@M (5 mg/kg) was injected intravenously into 8505C tumor-bearing BALB/c nude mice, Len-FITC was administered orally, and fluorescence imaging was performed at different time intervals after injection. Then, the mice were euthanized immediately to obtain the main organs and tumors for biological distribution analysis. Meanwhile, pharmacokinetic evaluation was conducted on fresh blood samples of equal volume using a fluorescence spectrophotometer. Three parallel samples were set for each group.

### In vivo synergistic therapeutic effect

The 8505C tumor-bearing BALB/c nude mice were randomly divided into 9 groups when the tumor volume reached 70 to 100 mm^3^ (*n* = 8 per group): (a) Saline, (b) US, (c) Len, (d) F, (e) F@M, (f) FL@M, (g) F + US, (h) F@M + US, and (i) FL@M + US. Aside from the fact that Len was administered orally, MOFs (5 mg/kg) were delivered via tail vein injection in mice on day 0, and US irradiation (1.5 W/cm^2^, 3 min) was performed 1 h after injection. The tumor volumes and body weights of the mice were measured every 3 days after treatment [tumor volume = (length × width^2^)/2]. Three mice from each group were randomly selected and sacrificed after 24 h following US irradiation, and tumor tissues were collected for H&E staining, terminal deoxynucleotidyl transferase-mediated deoxyuridine triphosphate nick end labeling (TUNEL) staining (Servicebio, Wuhan), Ki67 staining (Servicebio, Wuhan), and CD31 staining (ab28364, Abcam). After 21 days posttreatment, all mice were euthanized, and main organs and tumors were collected.

### In vivo inhibition of tumor recurrence

We used 8505C cells that overexpressed luciferase gene (8505C-luc) to establish a tumor-bearing mouse model. Briefly, 8505C-luc cells were implanted in the right armpit of mice. When the tumor grew to about 100 mm^3^, all mice were randomly divided into 3 groups (5 mice in each group): (a) Con (no treatment), (b) Sur (tumor resection surgery), and (c) Sur-SDT (surgery and postoperative SDT). In the Sur group, mice received resection surgery of tumor. For the Sur-SDT group, mice received surgery and postoperative SDT irradiated by US (1.5 mW/cm^2^) for 3 min every 3 days, with a total of 3 treatments. One day after SDT, mice were subjected to bioluminescent imaging.

### In vivo inhibition of tumor metastasis

To establish pulmonary metastasis models, 8505C cells (3 × 10^5^, 100 μl) were intravenously injected by tail vein. Starting from day 12 postinoculation, the mice received local SDT treatment every 3 days, with a total of 3 treatments. After monitoring for 14 days, mice were euthanized, and their lung tissues were collected for H&E staining.

### Statistical analysis

All the results were statistically analyzed by GraphPad Prism 8.0 software. The 2-tailed unpaired Student *t* test and one-way analysis of variance test were used to determine statistical significance (ns, *P* > 0.05; *P < 0.05; **P < 0.01; ***P < 0.001). The results were shown as mean ± SD.

## Results and Discussion

### Synthesis and characterization of FL@M

**Fig. 2.**
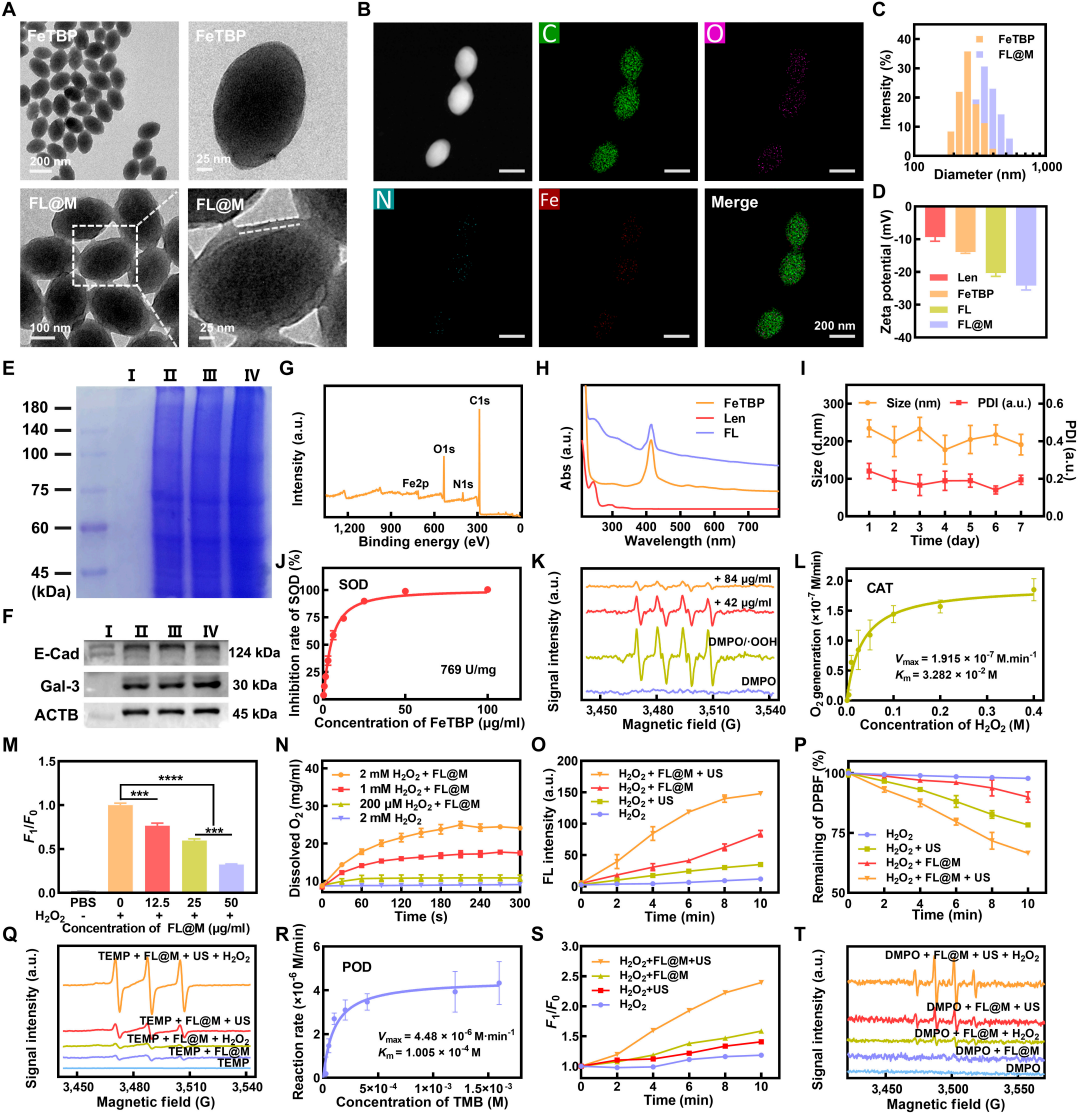
Synthesis and characterization of FL@M. (A) TEM of FeTBP and FL@M. (B) Element mapping for FeTBP. (C and D) Particle diameter size distribution (C) and zeta potential (D) of FeTBP, FL, and FL@M. (E) SDS-PAGE analysis of FL (I), FL@M (II), cancer cell membrane (III), and cell lysate (IV). (F) Western blotting analysis of E-cadherin, Galectin-3, and β-actin of FL(I), FL@M (II), cancer cell membrane (III), and cell lysate (IV). (G) XPS pattern of FeTBP. (H) UV–vis absorption spectra of Len, FeTBP, and FL. (I) The stability of FL in PBS (*n* = 3). (J) The SOD-like activity of FeTBP was measured by a commercial SOD assay kit in the WST manner. (K) The SOD activity of FL@M was detected by the ESR spectra of DMPO/O_2_^•−^. (L) The Michaelis–Menten fitting curve of dissolved oxygen generation rate versus H_2_O_2_ concentration in FeTBP. (M) H_2_O_2_ depleting ability of FL@M detected with ROS Green probe (*n* = 3). (N) The dissolved oxygen level of FL@M reacted with H_2_O_2_ detected by an oxygen sensor (*n* = 3). (O) Relative fluorescence intensity of DCFH-DA/total ROS at 525 nm with FL@M under US irradiation (*n* = 3). (P) ^1^O_2_ production by FL@M under different treatments detected by DPBF (*n* = 3). (Q) The ESR spectra of TEMP/^1^O_2_ for FL@M under US irradiation. (R) The Michaelis–Menten fitting curve of initial hydroxyl radical generation rate versus TMB concentration in FeTBP. (S) Relative fluorescence intensity of APF/•OH at 515 nm with FL@M under US irradiation (*n* = 3). (T) The ESR spectra of DMPO/•OH for FL@M under US irradiation. ***P < 0.001, **** *P <* 0.0001.

Firstly, FeTBP was synthesized with Fe_3_O clusters and 5,10,15,20-tetra(p-benzoate)porphyrin (H_4_TBP) ligand via the hydrothermal method [26]. Then, FL was obtained by loading Len and further modified with purified 8505C cell membrane fragments to obtain biomimetic nanoparticles FL@M (Fig. 1). TEM images revealed that FeTBP and FL@M nanoparticles were well-dispersed with a nanorice morphology (Fig. 2A). The elemental mapping showed that FeTBP contained C, N, O, and Fe, which coincided with XPS results (Fig. 2B and G and Fig. S1), and the iron content in FeTBP was determined to be 7.6089% by ICP-MS (Table S1). Moreover, there was a thin film surrounding the nanorice structure of FL@M, indicating the effective modification of cell membrane on FeTBP (Fig. 2A). In addition, the SDS-PAGE analysis presented that the protein profile of FL@M (II) was consistent with that of the purified cancer cell membrane (III), demonstrating the preservation of membrane protein (Fig. 2E). Western blotting analyses verified that the 2 membrane markers of E-cadherin and Galectin-3 were present on both the purified cell membrane and FL@M, further proving the successful modification of cell membrane (Fig. 2F). Besides, the average hydrated particle sizes of FeTBP and FL@M were 255 and 346 nm, respectively (Fig. 2C). Because of the overall electronegativity of the cell membrane, the zeta potential of FL@M was reduced to −26.0 mV compared with FL (−22.90 mV), which was lower than that of FeTBP (−12.93 mV) (Fig. 2D). The UV–vis spectroscopy result of FL was in accordance with the existence of absorption peaks of Len at 220 and 243 nm, indicative of the effective loading of Len (Fig. 2H). Moreover, the loading efficiency of Len positively correlated with the corresponding concentrations, and FL@M exhibited a good loading capacity of Len (77.55% ± 5.68%) at a dose of 1.5 mg (Figs. S2 and S3). Furthermore, we investigated the stability of FL, which is an important indicator of nanoparticles’ suitability for injection in vivo [38]. There were no discernible alterations in the size and polydispersity index (PDI) of FL in PBS during 1 week (Fig. 2I). The above results all demonstrated the successful synthesis of FL@M.

Additionally, we investigated the ability of FL@M to alleviate hypoxia. The superoxide dismutase (SOD)-like activity of FeTBP was quantitatively tested to be ∼769 U/mg by the disodium water-soluble tetrazolium (WST) method (Fig. 2J). Then, we used DMPO as an O_2_^•−^ capture agent to test the SOD-like activity of FL@M by ESR spectra. Compared to the DMPO group, the characteristic quadruple signal of O_2_^•−^/DMPO decreased substantially with the concentration of FL@M, suggesting that FL@M can effectively convert O_2_^•−^ into H_2_O_2_ and O_2_ (Fig. 2K). Moreover, the CAT-like activity of FeTBP was evaluated by the dissolved O_2_ generation method, and the parameter indicators were calculated using Michaelis–Menten kinetics (*K*_M_ = 32.82 mM and *V*_max_ = 1.915 × 10^−4^ mM/min) (Fig. 2L). Meanwhile, the CAT-like activity of FL@M was tested by ROS Green probe and dissolved oxygen analyzer, respectively. The results indicated that FL@M had strong CAT-like activity (Fig. 2M and N).

Furthermore, the total ROS production of FL@M was investigated by DCFH-DA, whose fluorescence intensity at 525 nm gradually increased with the progress of the reaction (Fig. 2O and Fig. S4). FL@M could stably generate more cytotoxic ROS under US irradiation and H_2_O_2_,markedly higher than that with only US or H_2_O_2_ (Fig. 2O). In addition, the UV–vis absorption of DPBF declined gradually with time and the FL@M + H_2_O_2_ + US group showed the most noticeable decrease, indicating that FL@M could effectively generate ^1^O₂ under US irradiation (Fig. 2P and Fig. S5). Meanwhile, the ESR spectrum of FL@M presented a characteristic 1:1:1 triplet signal of TEMP/^1^O₂ and the most obvious signal was observed in the TEMP + FL@M + US + H_2_O_2_ group (Fig. 2Q). Besides, the POD-like activity result of FeTBP demonstrated that the absorbance value of the mixture solution at 652 nm increased continuously as the reaction proceeded (Fig. 2R). As probe of •OH, the fluorescence intensity of APF rose in the FL@M + H_2_O_2_ group, indicating that FL@M could generate •OH through Fenton reaction, and •OH production was enhanced in the presence of H_2_O_2_ and US irradiation, indicating its sonodynamic effect (Fig. 2S). Moreover, the ESR result of FL@M showed that the characteristic signal of DMPO/•OH under H_2_O_2_ and US irradiation could magnify •OH production of FL@M, confirming the similar tendency with APF (Fig. 2T). Taken together, FL@M could not only alleviate hypoxia by producing O_2_ through SOD and CAT-like activity, but also exhibit good SDT efficiency, including ^1^O₂ and •OH production.

### Intracellular uptake and distribution of FL@M

**Fig. 3.**
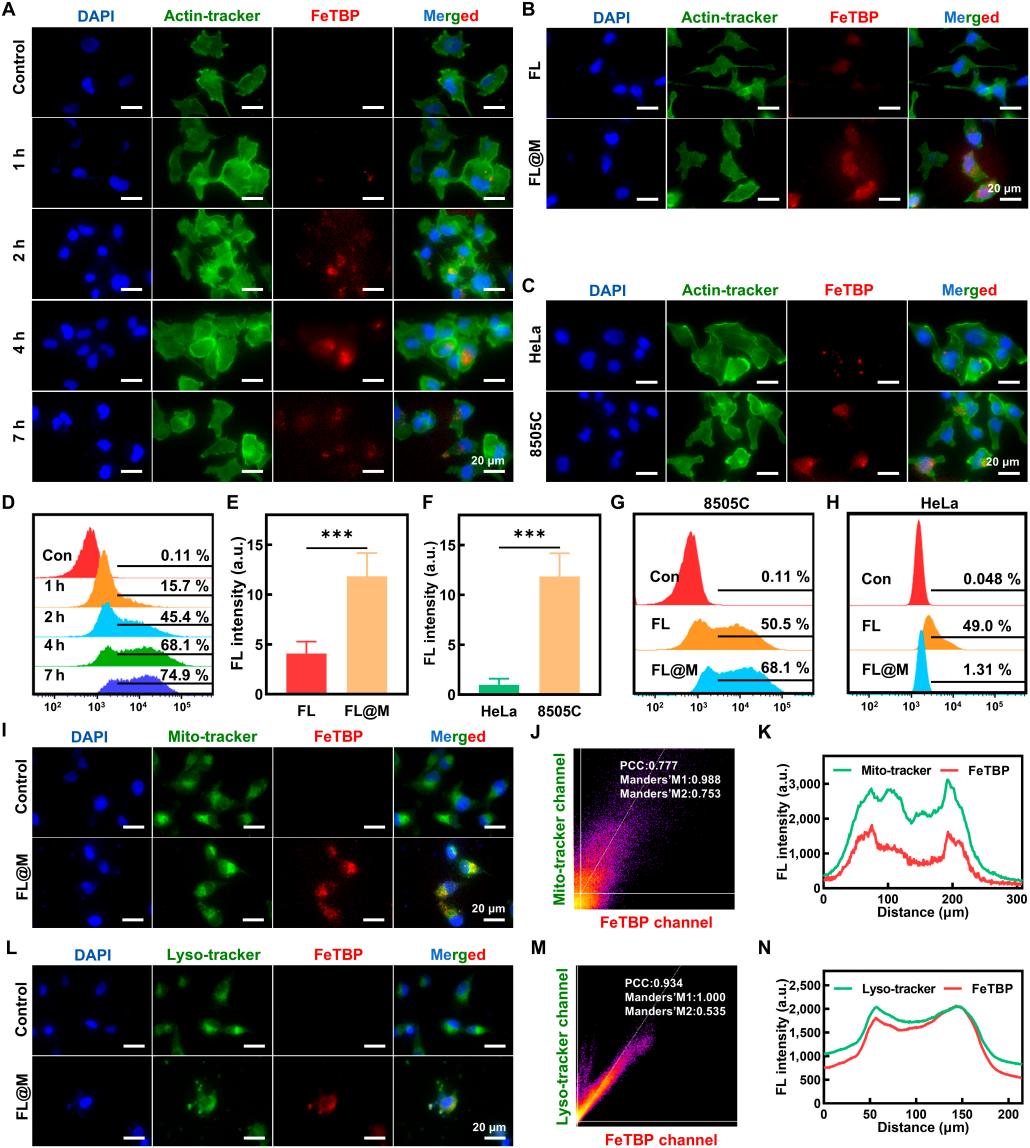
Cellular uptake and intracellular distribution of FL@M. (A) Representative fluorescence images of 8505C cells incubated with FL@M at different times. (B) Representative fluorescence images of 8505C cells incubated with FL or FL@M at 4 h. (C) Representative fluorescence images of HeLa cells and 8505C cells incubated with FL@M at 4 h. (D) Flow cytometry of 8505C cells incubated with FL@M at different times. (E) Quantification of fluorescence intensity of FeTBP shown in (B). (F) Quantification of fluorescence intensity of FeTBP shown in (C). (G) Flow cytometry of 8505C cells incubated with FL or FL@M at 4 h. (H) Flow cytometry of HeLa cells incubated with FL or FL@M at 4 h. (I) Representative colocalization fluorescence images of FL@M and mitochondria. FeTBP fluorescence (red), Mito-Tracker Green (detect mitochondria), and DAPI (blue). (J) The Pearson’s correlation coefficient (PCC) and Mander’s correlation coefficients (MCC) analysis of FL@M with mitochondria. (K) Plot profile analysis of Mito-Tracker colocalization with FL@M. (L) Representative colocalization fluorescence images of FL@M and lysosomes. FeTBP fluorescence (red), Lyso-Tracker Green (detect lysosome), and DAPI (blue). (M) The PCC and MCC analysis of FL@M with lysosome. (N) Plot profile analysis of Lyso-Tracker colocalization with FL@M. ***P < 0.001.

As a simple and effective biomimetic strategy, cell membrane-camouflaged nanoparticles can maintain their membrane structure and antigens, achieving special functions such as prolonging blood circulation time, immune escape, and specific recognition [39,40]. Therefore, we assessed the targeting uptake efficiency of FL@M in ATC cells. To ascertain the uptake efficiency and the optimal time for US irradiation in vitro, we detected the intracellular fluorescence intensity of FeTBP at various incubation times. A faint red fluorescence signal was observed in 8505C cells incubated with FL@M for 1 h (Fig. 3A). As time increased, the red fluorescence signal gradually strengthened and reached the peak at 4 h (Fig. 3A). The flow cytometry results indicated that the red fluorescence signal was obviously high in 4 h (Fig. 3D), suggesting that the intracellular accumulation of FL@M was in a time-dependent way and we choose 4 h as the incubation time for subsequent experiments.

Next, we evaluated the targeting capability of FL@M. The images showed that the red fluorescence intensity of the cell membrane-modified group (FL@M) was stronger than that of the unmodified group (FL), revealing that FL@M had good targeting ability toward 8505C cells (Fig. 3B and E). Similar results were observed in the flow cytometry results (Fig. 3G). Moreover, HeLa cells incubated with FL@M (8505C cells membrane-modified) showed weak red fluorescence intensity compared with the FL group (Fig. 3H and Fig. S6). Additionally, the red fluorescence was significantly stronger in the cytoplasm of 8505C cells than HeLa cells after 4 h incubation with FL@M (8505C cells membrane-modified), providing evidence that the biomimetic camouflage membrane enhances specific self-recognition (Fig. 3C and F). Meanwhile, the flow cytometry results of FL@M encapsulating HeLa cell membrane also supported that the uptake rate of nanoparticles was obviously increased in homologous cells (HeLa) while substantially decreased in nonhomologous cells (8505C) (Fig. S7). In summary, biomimetic nanoreactors coated with cancer cell membranes demonstrated excellent homologous targeting ability for 8505C cells.

Then, we further evaluated targeted drug delivery ability using Len-FITC. The flow cytometry results exhibited that the cellular uptake of Len in the FL@M group was higher than that of the Len group, indicating that the nanocarrier improved the delivery of Len (Fig. S8). Moreover, according to the results of the fluorescence imaging, the green fluorescence of Len-FITC partially overlapped with the red fluorescence signal of FeTBP after 4 h incubation while this colocalization decreased after US irradiation, indicating that Len can be released from FL@M under US irradiation (Fig. S9).

Additionally, we determined the intracellular distribution of FL@M in 8505C cells. Fluorescence imaging revealed that the red fluorescence signal of FL@M partially overlapped with the green fluorescence of Mito-Tracker probe, indicating that FL@M mainly distributed on mitochondria (Fig. 3I to K). Similarly, FL@M was found to colocalize with Lyso-Tracker Green, supporting that nanocarriers were also partially trapped in lysosomes (Fig. 3L to N). These findings demonstrated that FL@M was located in mitochondria and lysosomes after absorption.

### Therapeutic efficacy of FL@M in vitro

**Fig. 4.**
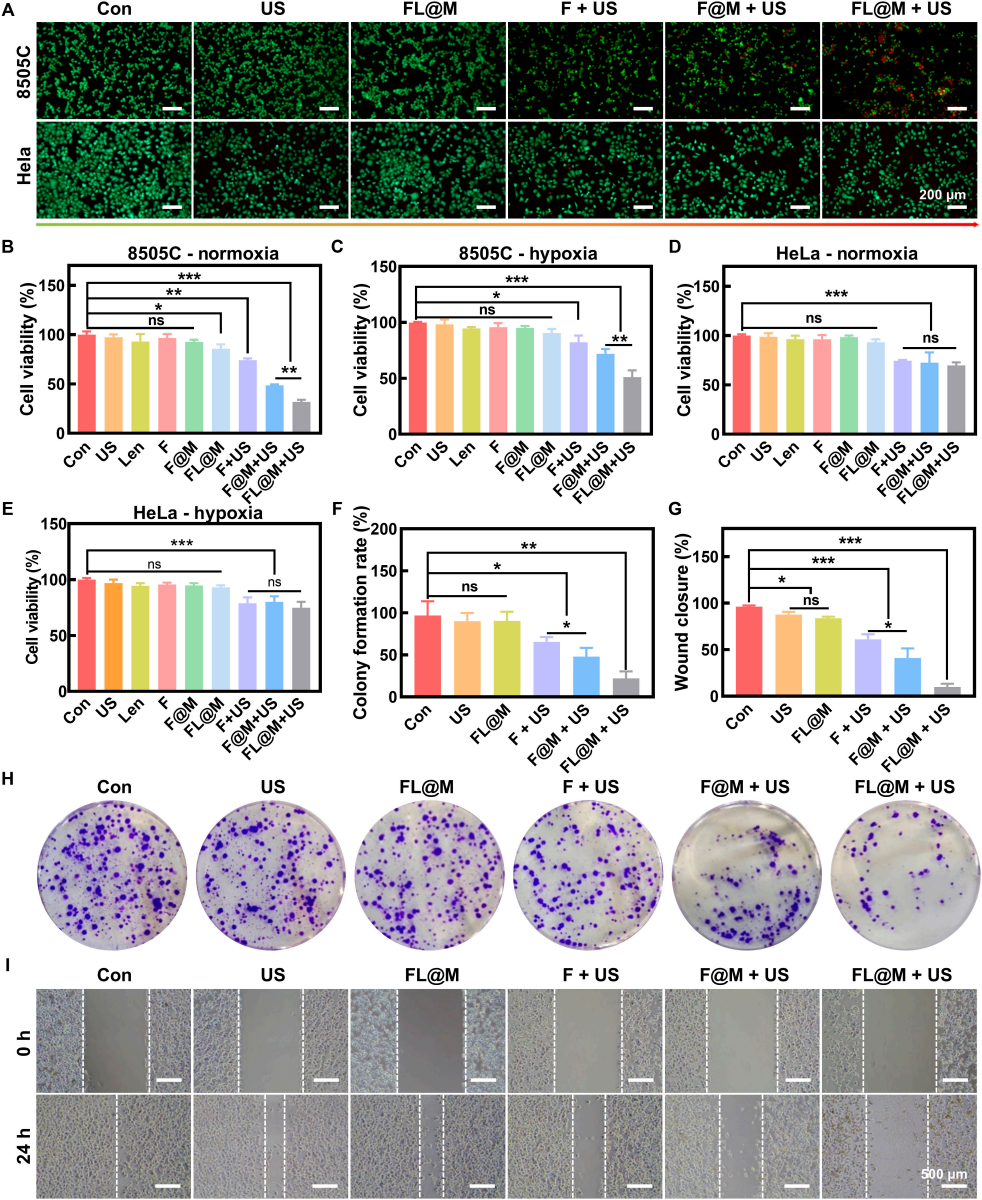
FL@M inhibits tumor proliferation and metastasis. (A) Calcein-AM/PI assay fluorescence images of 8505C and HeLa cells after different treatments. (B and C) 8505C cells’ viabilities following various treatments under normoxia (B) and hypoxia (C) detected by MTT (*n* = 3). (D and E) HeLa cells’ viabilities following various treatments under normoxia (D) and hypoxia (E) detected by MTT (*n* = 3). (F and H) Quantification of clonogenic assays (F) and representative images (H) for 8505C cells after various treatments (*n* = 3). (G and I) Quantification of wound closure (G) and representative images (I) after different treatments in 8505C cells (*n* = 3). *P < 0.05, **P < 0.01, and ***P < 0.001.

First, the cytotoxic effects of FL@M on 8505C cells were assessed by Calcein-AM/PI staining (Fig. 4A). The cell mortality substantially increased in SDT-treated 8505C cells, which showed markedly reduced green fluorescence intensity and increased red fluorescence intensity compared with control groups. As expected, the best therapeutic efficacy was observed in the FL@M combined US group of 8505C cells. In contrast, there was no obvious cell death in each group of HeLa cells (Fig. 4A), attributing to the cell membrane camouflage targeting to 8505C cells.

Meanwhile, the therapeutic efficacy of FL@M on 8505C cells and HeLa cells was also assessed by MTT assay. The results showed that the viability of 8505C cells was slightly affected by only US, Len, or FL@M under both normoxic and hypoxic conditions. In contrast, 8505C cells showed decreased viabilities after US irradiation, especially in the FL@M + US group (Fig. 4B and C). In HeLa cells, FL@M coated with 8505C cell membrane-based SDT treatment showed a mild killing effect (Fig. 4D and E). Moreover, FL@M exhibited a better cell-killing effect than the Len group at the same concentration (Fig. S10). Together, these results supported the targeting ability of homologous tumor cell membranes and proved that FL@M could exhibit effective SDT under both normoxic and hypoxic conditions.

In addition, cell cloning assay was conducted to evaluate the effect of FL@M on the proliferation capacity of 8505C cells. The colony formation rate of 8505C cells was lower in SDT treatment groups (F + US, 65.61% ± 5.67% and F@M + US, 47.92% ± 9.50%) compared to the US group (90.19% ± 9.73%) and FL@M group (90.53% ± 10.77%). The lowest colony formation rate was observed in the FL@M + US group (20.14% ± 8.31%) (Fig. 4F and H). Edu assay also confirmed the same results (Fig. S11). Furthermore, the cell scratch tests revealed that the FL@M + US group had the largest scratch area (9.92% ± 3.47%) among all groups (CON, 96.28% ± 1.24%; US, 87.66% ± 3.02%; FL@M, 83.73% ± 1.75%; F + US, 61.10% ± 5.55%; F@M + US, 41.09% ± 10.25%), indicating significant inhibition of cell migration ability (Fig. 4G and I). Similar results were found in transwell migration assay (Fig. S12). In summary, FL@M exerted a strong synergistic therapeutic effect on inhibiting the proliferation and migration of thyroid cancer cells.

### FL@M effectively enhances ROS generation and induces cell apoptosis

**Fig. 5.**
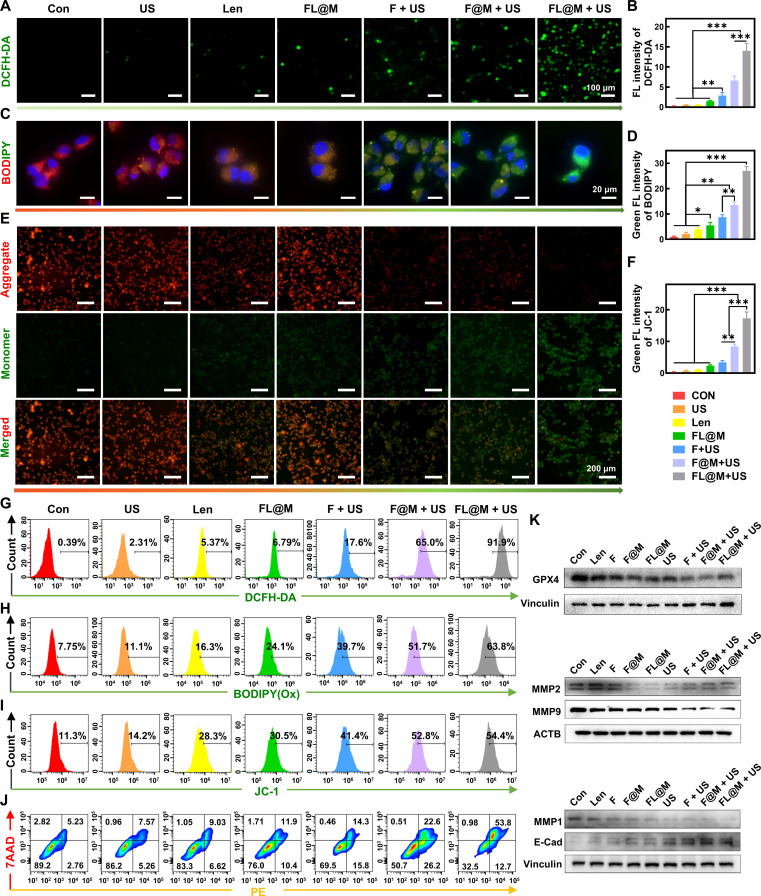
FL@M enhances oxidative stress and induces apoptosis. (A) ROS production in 8505C cells after different treatments was detected by DCFH-DA. (B) Quantification of intracellular fluorescence intensity of DCFH-DA shown in (A). (C) Lipid oxidation levels in 8505C cells after different treatments were detected by BODIPY. (D) Quantification of intracellular fluorescence intensity of lipid oxidation level shown in (C). (E) The changes in the MMP of 8505C cells after different treatments were detected by JC-1. (F) Quantification of intracellular fluorescence intensity of MMP shown in (E). (G) Flow cytometry was used to assess ROS production after different treatments detected by DCFH-DA. (H) Flow cytometry was used to assess lipid oxidation levels after different treatments detected by BODIPY. (I) Flow cytometry was used to assess MMP after different treatments detected by JC-1. (J) Apoptosis of 8505C cells was assessed by flow cytometry with Annexin V-PE/7AAD staining. (K) Western blotting analysis of GPX4, MMP2, MMP9, MMP1, and E-Cad protein in 8505C cells with various treatments. *P < 0.05, **P < 0.01, and ***P < 0.001.

Sonosensitizers enriched in tumor sites can generate a substantial amount of ROS under US irradiation, inducing oxidative stress and ultimately killing cells [41]. Based on the comprehensive therapeutic effect on 8505C cells, we further evaluated the ROS generation ability of FL@M by DCFH-DA. The weak fluorescence signal was observed in the Len group and FL@M group, slightly higher than the US group and control group. In contrast, MOFs resulted in pronounced green fluorescence signals after US irradiation, particularly in the FL@M + US group (Fig. 5A and B). The flow cytometry results were consistent with the microscopic images (Fig. 5G). Moreover, hypoxia environment is an important factor in limiting SDT effectiveness, so we investigated the ability of FL@M to alleviate hypoxia. Intracellular O_2_ level and H_2_O_2_ content were assessed by RDPP probe and ROS Green probe, respectively. Flow cytometry results proved that FL@M could effectively decrease the fluorescence signal of RDPP and ROS Green in hypoxic conditions, exhibiting that FL@M could decompose H_2_O_2_ into O_2_ through CAT-like ability (Figs. S13 and S14). Moreover, the expression of HIF-1α protein was investigated using Western blotting, and it showed that FL@M substantially reduced the HIF-1α levels under hypoxia. (Fig. S15). In conclusion, the above data demonstrated that FL@M could effectively increase ROS production and alleviate tumor hypoxia.

The cell membrane and organelle membranes contained a large amount of polyunsaturated fatty acids, which were particularly susceptible to ROS damage [42,43]. The Fenton reaction enhanced the production of •OH, which preferentially reacted with tumor cell membranes and triggered lipid peroxidation [44]. Therefore, we employed C11-BODIPY581/591 as an intrinsically lipophilic fluorescent indicator to assess lipid peroxidation levels [45]. As shown in results, the Len-only group showed moderate oxidative stress, and when combined with SDT, the FL@M + US group exhibited the most pronounced green fluorescence, aligned with ROS levels, indicating severe oxidative damage to cell membranes (Fig. 5C, D, and H).

Mitochondria served as the primary site for ROS generation and were prone to lipid peroxidation damage. To investigate the mechanism underlying FL@M-mediated SDT cell killing, the MMP of 8505C cells was detected by JC-1 probe. The decrease in MMP was characterized by increased green fluorescence, indicating mitochondrial dysfunction and early apoptotic events [46]. According to the results, cells in the US group and the FL@M group showed a slight green fluorescence (Fig. 5E). Following US irradiation, nanoparticles resulted in significantly green fluorescence signals, showing a substantial loss of MMP (Fig. 5E and F). These findings were further corroborated by the flow cytometry results (Fig. 5I). Therefore, we speculated that a large amount of ROS generated by SDT induced lipid peroxidation and mitochondrial dysfunction.

In addition, the cell death mechanism was further examined through Annexin V-PE/7AAD double staining assays. The flow cytometry results showed that the SDT group’s cells induced more apoptosis than the control group. The cell apoptosis rate of the FL@M + US group reached as high as 66.5%, markedly higher than that of other groups (F + US, 30.1% and F@M + US, 48.8%), demonstrating that Len and SDT combination therapy effectively induced cell apoptosis (Fig. 5J). Western blotting results showed that compared with other groups, the SDT groups substantially reduced the expression of GPX4, MMP-1, MMP-2, and MMP-9, while increasing E-cadherin. This further confirmed that FL@M-SDT could reduce intracellular antioxidant capacity and simultaneously inhibit the invasive abilities of cells (Fig. 5K). Consequently, the above results demonstrated that FL@M-mediated SDT achieved a synergistic killing effect and effectively induced apoptosis on 8505C cells.

### Biocompatibility of FL@M in vivo

The biosafety of FL@M both in vitro and in vivo was evaluated. First, hemocompatibility results showed that FL@M exhibited negligible hemolytic activity (<5%) across a broad concentration range (100 to 1,000 μg/ml) (Fig. S16). Furthermore, healthy male KM mice were randomized into 4 groups, with the experimental groups receiving FL@M (5 mg/kg) or saline via tail vein injection and observation for 14 and 30 days, respectively. During administration and observation, the weight of the mice showed an upward trend with no significant difference between the groups (Fig. S17). Then, all mice were sacrificed to harvest blood and important organs for examination. According to the results of the blood routine examination, there were no significant differences in RBCs, white blood cells, hemoglobin, platelets, and lymphocytes among groups (Fig. S18). The biochemistry results showed no significant difference in the renal and liver function (Fig. S19). In addition, H&E staining results presented that almost no pathological damage were observed in the hearts, livers, spleens, lungs, and kidneys of mice (Fig. S20). Based on the above results, FL@M exhibited outstanding biocompatibility.

### Targeting ability, biodistribution, and pharmacokinetics of FL@M in vivo

**Fig. 6.**
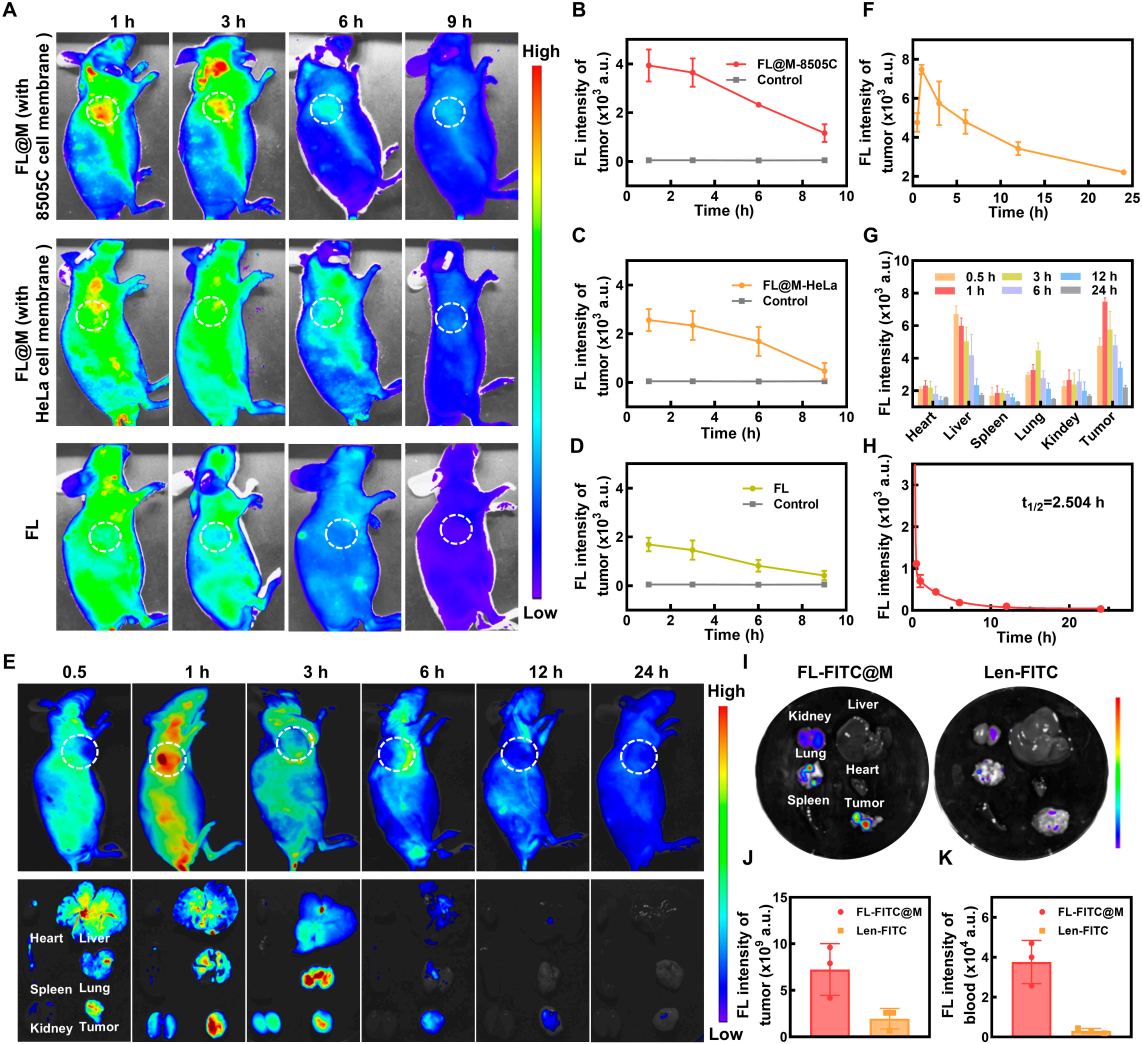
In vivo biodistribution, and pharmacokinetics of FL@M. (A) Representative fluorescence images of 8505C tumor-bearing mice at different times after injection of FL or FL@M (coated with 8505C cell membrane or HeLa cell membrane). (B) The fluorescence intensity of tumors changed over time after intravenous injection of FL@M coated with homologous cell membrane 8505C cell membrane in (A) (*n* = 3). (C) The fluorescence intensity of tumors changed over time after intravenous injection of FL@M coated with heterologous HeLa cell membrane in (A) (*n* = 3). (D) The fluorescence intensity of tumors changed over time after intravenous injection of FL (no targeted modification) in (A) (*n* = 3). (E) Representative fluorescence images at different time points of mice, vital organs, and tumors after injection of FL@M coated with homologous cell membrane (8505C cell membrane). (F) The fluorescence intensity of tumors changes over time in (E) (*n* = 3). (G) The fluorescence intensity of vital organs at different time points in (E) (*n* = 3). (H) Pharmacokinetics of FL@M in 8505C tumor-bearing mice (*n* = 3). (I) Representative fluorescence images of vital organs and tumors at 1 h after oral administration of Len-FITC or intravenous injection of FL-FITC@M (coated with 8505C cell membrane). (J) The fluorescence intensity of tumors in (I) (*n* = 3). (K) The fluorescence intensity of Len in serum of 8505C tumor-bearing mice (*n* = 3).

Building upon the outstanding biosafety of FL@M, the self-targeting ability toward thyroid tumors was further investigated in nude mice bearing subcutaneous tumors. The fluorescence signal of FL@M was captured at different time points after intravenous administration of mice, and the fluorescence intensity at the tumor site was gradually decreased with time (Fig. 6A to D). Meanwhile, FL@M coated with homologous cell membrane had markedly higher aggregation at the tumor site than FL@M coated with heterologous cell membrane and the FL group (Fig. 6A). The above results indicated that biomimetic nanoreactors coated with cancer cell membranes exhibited excellent homologous ability through the specific self-targeting of cancer cells.

Next, we explored the biodistribution of FL@M. The fluorescence imaging results showed that the fluorescence signal appeared at the tumor sites as soon as 30 min and peaked at 1 h after injection, suggesting efficient accumulation of nanoparticles in subcutaneous tumors, supporting efficient tumor uptake of FL@M. Moreover, only weak fluorescence signals were detected in subcutaneous tumors after 24 h (Fig. 6E and F). Meanwhile, the fluorescence imaging and quantitative analysis results of major organs and tumors showed that FL@M was mainly distributed in the tumor, liver, kidneys, and lungs, and the distribution of FL@M at tumor sites was substantially greater than that in other organs (Fig. 6E and G). According to the pharmacokinetic study, the blood fluorescence intensity gradually decreased postinjection, with a half-life (*t*_1/2_) of 2.504 h, revealing that FL@M could effectively metabolize and be eliminated from the body (Fig. 6H).

To determine the drug delivery capacity of FL@M in vivo, FL@M that contained Len-FITC was further explored in nude mice bearing subcutaneous tumors. Upon 1 h of oral administration of Len-FITC or intravenous injection of FL-FITC@M, tumors and vital organs were acquired for ex vivo analysis. We found that the FL-FITC@M group exhibited markedly higher tumor enrichment compared to the oral Len-FITC group, indicating that biomimetic nanoparticles could deliver more Len into the tumor microenvironment (Fig. 6I and J). Meanwhile, the fluorescence intensity of FITC in the serum in the FL-FITC@M group was significantly higher than that in the oral Len-FITC group (Fig. 6K). In summary, these results exhibited that FL@M had excellent tumor-targeting capability for drug delivery, a favorable biodistribution profile, and efficient metabolic clearance, supporting its application of tumor treatment in vivo.

### Synergistic therapeutic efficacy of FL@M in vivo

**Fig. 7.**
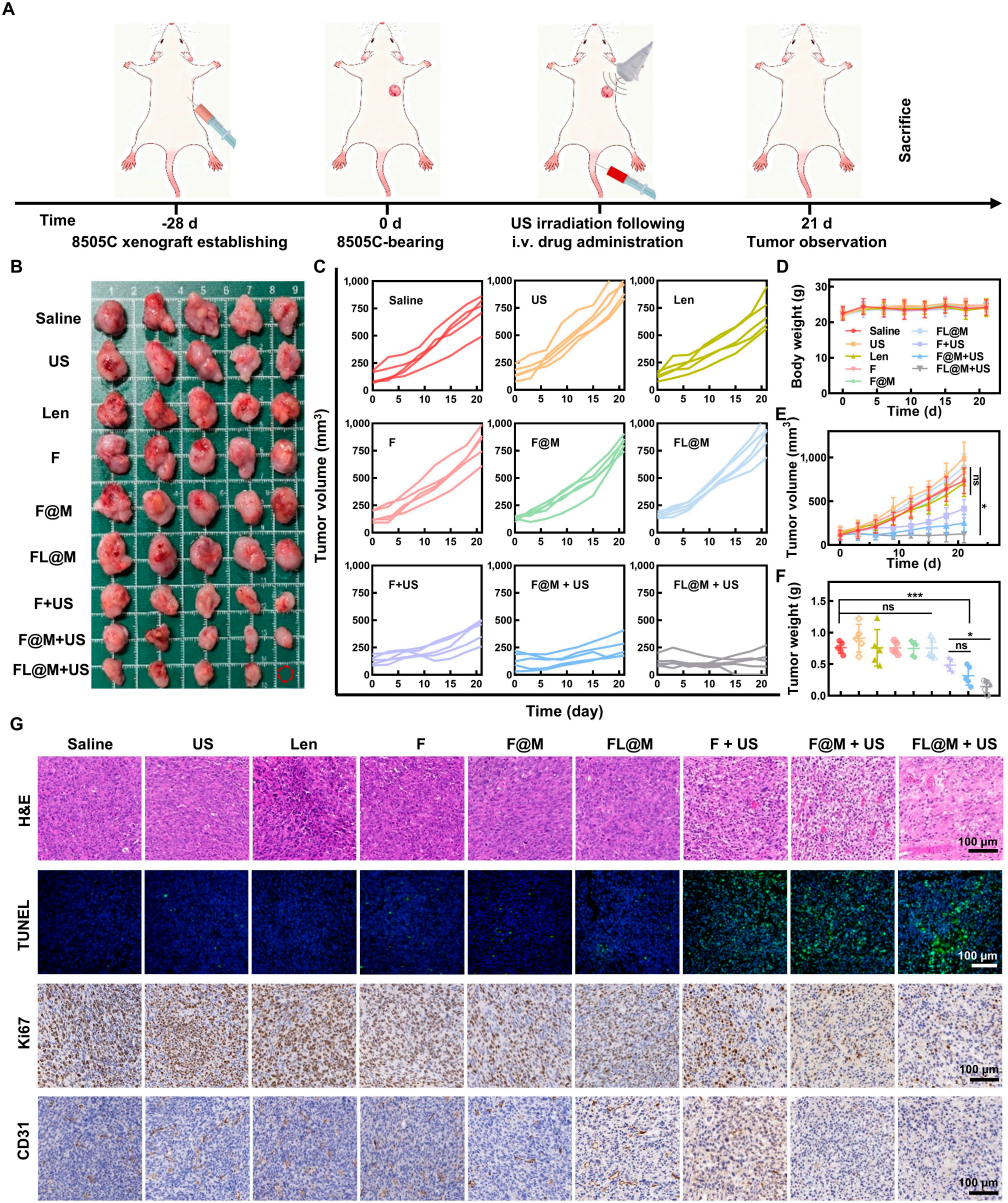
In vivo synergistic therapeutic efficiency of FL@M on 8505C tumor-bearing mice. (A) Schematic diagram of the experimental design. (B) Photograph of tumors for all treated groups. (C) The change of tumor volume under various treatments (*n* = 5). (D) The change of body weight under different treatments (*n* = 5). (E) The change of tumor volume under different treatments (*n* = 5). (F) Tumor weight under various treatments was measured (*n* = 5). (G) Pictures of tumor tissue stained with H&E, TUNEL, Ki-67 and CD31. *P < 0.05 and ***P < 0.001.

The therapeutic efficacy of FL@M on tumors was further validated in vivo. The BALB/C nude mice were randomly divided into 9 treatment groups when the tumor volume reached 70 to 100 mm^3^: (a) Saline, (b) US, (c) Len, (d) F, (e) F@M, (f) FL@M, (g) F + US, (h) F@M + US, and (i) FL@M + US. According to the treatment plan, different nanoparticles (5 mg/kg) were injected into the mice via tail vein, and US irradiation (1.5 W/cm^2^, 3 min) was performed 1 h after injection (Fig. 7A). The body weights and tumor volumes of the mice were monitored every 3 days during the treatment period, and the tumor tissues were weighed at the end of the experiment. There were no obvious differences in body weight among all groups of mice (Fig. 7D), confirming the biocompatibility of FL@M. Meanwhile, 3 SDT groups showed significant growth inhibition in tumor volume compared to others. The slowest tumor volume progression and the lightest tumor weight were observed in the FL@M + US group, proving the superior efficacy (Fig. 7B, C, E, and F). In conclusion, these findings demonstrated that combined SDT/chemotherapy provided excellent treatment effects on thyroid cancer.

To assess the therapeutic impact of FL@M on thyroid cancer, tumor tissue of different treatment groups was observed by H&E staining. The tumor tissue of the FL@M group showed mild tissue damage with interstitial loosening and sparse gaps (Fig. 7G). There were more obvious conspicuous karyopyknosis and nuclear destruction in tumor tissue of the FL@M + US group compared to other groups, representing the shrinking of tumor cells and extensive destruction of tumor tissue (Fig. 7G). Meanwhile, the immunohistochemistry assay results demonstrated that the expression level of Ki-67 in the SDT groups decreased to varying degrees with the most obvious suppression occurring in the FL@M + US group (Fig. 7G). Besides, TUNEL staining showed similar results, revealing that the FL@M effectively inhibited tumor proliferation and induced tumor apoptosis (Fig. 7G). In addition, the FL@M + US group greatly weakened the expression of CD31 in tumor tissue, supporting the severe damage to vascular integrity and nutrition supply (Fig. 7G). In summary, FL@M has shown outstanding effects in inhibiting the proliferation of cancer cells and inducing cell apoptosis.

### FL@M inhibits tumor recurrence and metastasis in vivo

**Fig. 8.**
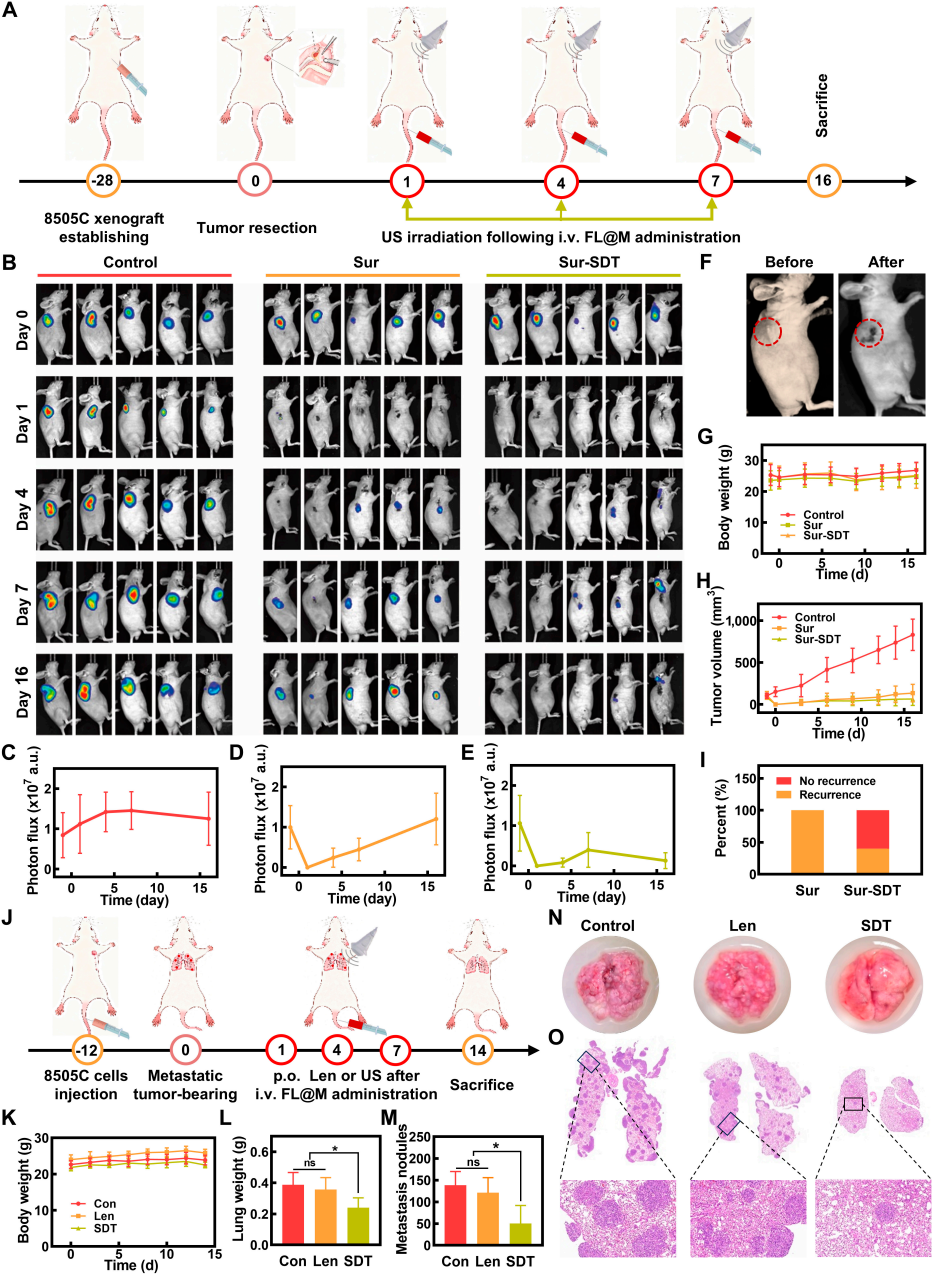
Therapeutic efficacy of FL@M in recurrent and metastatic tumor models. (A) Schematic illustration of surgery–SDT for recurrent tumors. (B) Representative bioluminescence images were performed to monitor tumor recurrence after the treatment in all mice. (C) The bioluminescence intensity of tumors in the control group at different times shown in (B). (D) The bioluminescence intensity of tumors in the surgery group at different times shown in (B). (E) The bioluminescence intensity of tumors in the surgery + SDT group at different times shown in (B). (F) Photos of 8505C tumor-bearing mice before and after surgery. (G) The weight records of recurrent 8505C tumor-bearing mice under different treatments (*n* = 5). (H) Average tumor growth curves of recurrent 8505C tumor-bearing mice under various treatments. (I) Tumor recurrence rate of recurrent 8505C tumor-bearing mice under different groups. (J) Schematic illustration of SDT for metastatic tumors. (K) The body weight of mice in 8505C metastatic tumor under different treatments (*n* = 5). (L) Lung weight of mice under various treatments was measured (*n* = 5). (M) The number of metastasis nodules in lung under various treatments was measured (*n* = 5). (N) Photos of lung for all metastatic tumors groups. (O) Pictures of lung tissue stained with H&E. *P < 0.05.

Research has shown that about 2/3 of thyroid cancer patients still experience tumor recurrence or metastasis after undergoing surgery and adjuvant RAI therapy [47], which is a main cause of treatment failure for patients. Therefore, to investigate whether FL@M SDT could inhibit tumor recurrence and metastasis, we constructed the recurrent tumor model by surgical resection of subcutaneous tumors in 8505C-luc tumor-bearing mice [48]. Mice were randomly divided into 3 groups (*n* = 5): control group (mice received no treatment), Sur group (mice received tumorectomy), and Sur–SDT group (mice received surgery and postoperative SDT) (Fig. 8A). Tumor growth was monitored by both in vivo imaging (Fig. 8B) and caliper measurement (Fig. 8H). In vivo imaging showed that the tumor volume of the control group increased as the time elapsed (Fig. 8C). In contrast, the tumor volumes were markedly decreased in the Sur and Sur–SDT groups after mice received surgery (Fig. 8D and E). The comparison of mice before and after surgical resection is shown in Fig. 8F. The body weights of mice were monitored during the treatment period (Fig. 8G). However, all mice experienced tumor recurrence in the Sur group on day 16. In contrast, only 2 of 5 mice in the Sur–SDT group experienced relapsed tumor, confirming the satisfactory therapeutic effect of Sur–SDT treatment to inhibit tumor recurrence (Fig. 8E and I).

We further investigated the therapeutic effect of FL@M on a metastatic tumor model. After intravenous injection of 8505C cells for 12 days, the mice were randomly divided into 3 groups: control group, Len group, and SDT group. The control group underwent injection of saline, while the Len group received an oral administration of Len, and the SDT group received US irradiation following the injection of FL@M every 3 days for 3 times, respectively (Fig. 8J). The body weight of mice in each group was measured at regular intervals throughout the treatment period (Fig. 8K). After treatment, all mice were euthanized and lung tissues were collected. There were increased lung tissue weights in the control and Len group (Fig. 8L). As shown in results, FL@M presented a marked reduction in surface metastatic nodules in lung tissue compared to the control group and Len group (Fig. 8M and N). In contrast, there was a significant reduction in lung metastases and weight in the SDT group while Len treatment showed no therapeutic effect compared with the control group (Fig. 8L and M). Meanwhile, H&E staining of whole lung lobes further confirmed a greater number and a larger size of lung metastatic foci in the control group and Len group (Fig. 8O). The above results reasonably indicated that FL@M-mediated SDT/Len combination therapy had good therapeutic effects on recurrent and metastatic tumors, bringing great hope for clinical application in thyroid cancer.

## Conclusion

In this work, we designed and synthesized an intelligent biomimetic nanocarrier based on iron-MOF by encapsulating Len for synergistic chemotherapy and SDT of thyroid cancer. The FL@M nanocarrier demonstrated excellent targeted cellular uptake and favorable biosafety profiles both in vitro and in vivo. Owing to its homologous cancer cell membrane coating, FL@M promoted specific internalization into 8505C cells. After absorption, FL@M alleviated tumor hypoxia and augmented ROS generation through iron-based enzyme activities, thereby potentiating the SDT efficacy. Furthermore, under US irradiation, FL@M enhanced Len release, initiated SDT and Len combination therapy, and finally induced intracellular oxidative stress, leading to pronounced tumor apoptosis. Collectively, the biomimetic nanocarrier FL@M represents a safe and effective MOF-based nanoplatform to inhibit tumor proliferation, recurrence, and metastasis, offering a promising SDT/chemotherapy combination strategy for thyroid cancer.

## Data Availability

The datasets used and analyzed during the current study available from the corresponding author on reasonable request.
