# Supplementary material for: Versatile Nanotherapeutics for Enhancing Sonodynamic Therapy/Chemotherapy of Thyroid Cancer through Remodeling Tumor Microenvironment and Synergistic Reactive Oxygen Species Augment
Source: Biomater Res. 2026 Mar 4;30:0338. doi: 10.34133/bmr.0338 (PMC12957540; doi:10.34133/bmr.0338)

**Supporting Information**


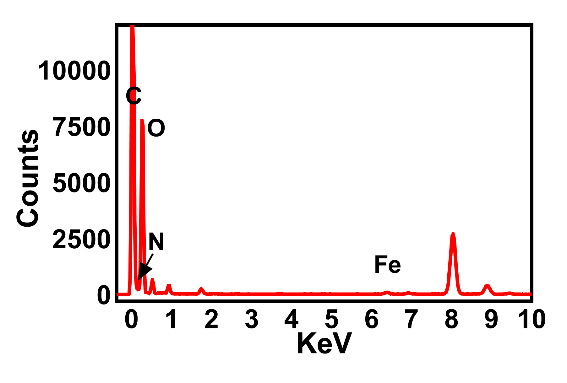


**Figure S1. The EDS element mapping spectrum of FeTBP.**


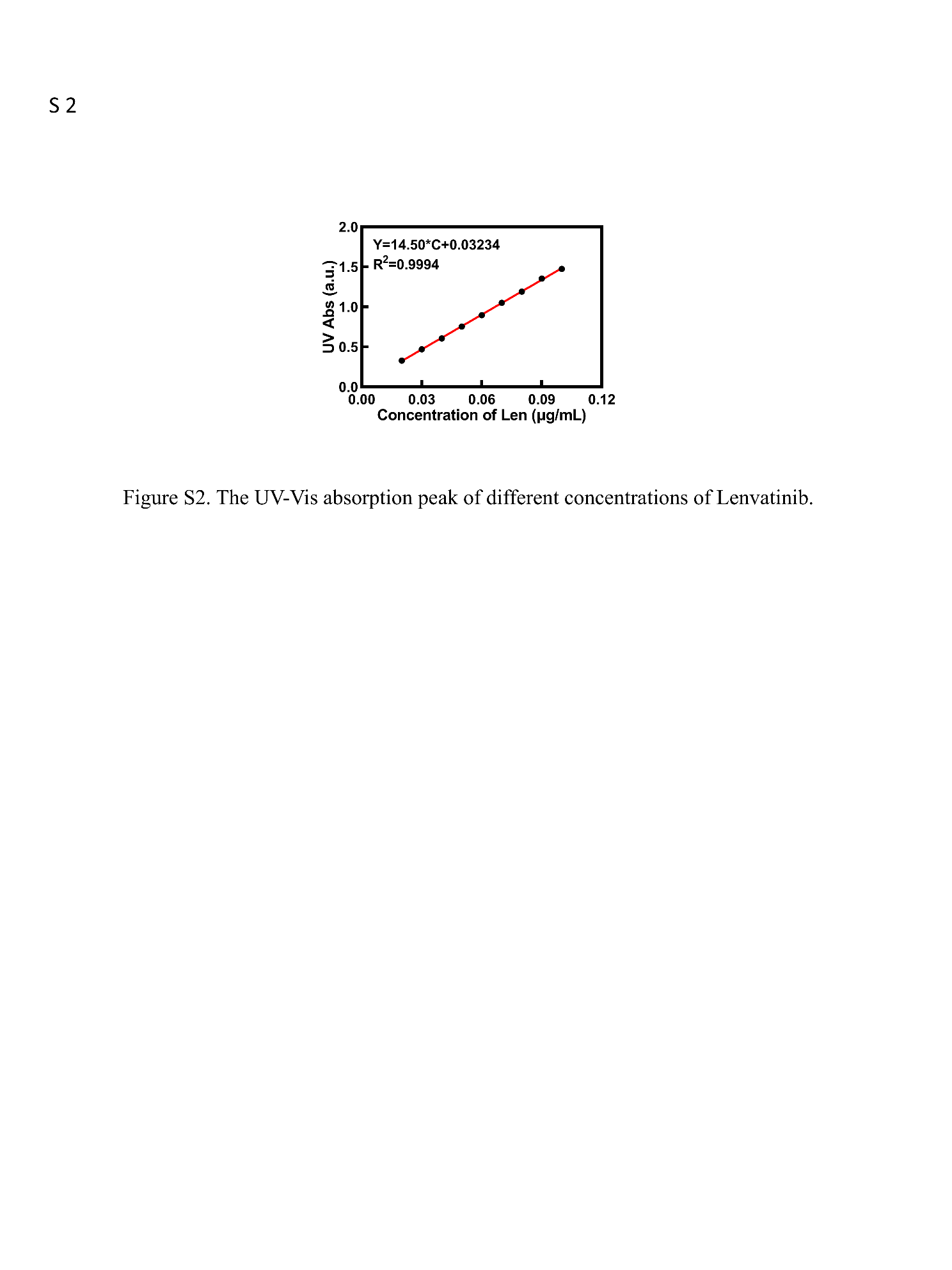


**Figure S2. The UV-vis absorption peak of different concentrations of Lenvatinib.**


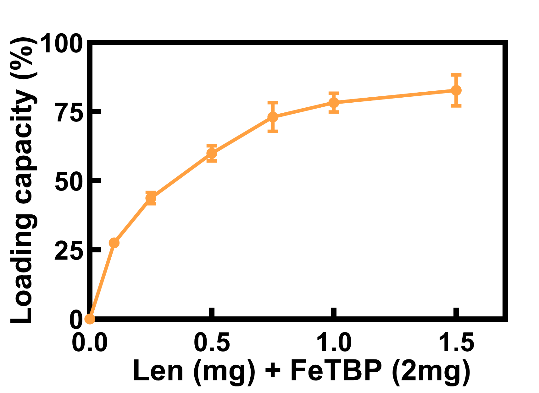


**Figure S3. The loading efficiency of Len in FL@M (n = 3).**


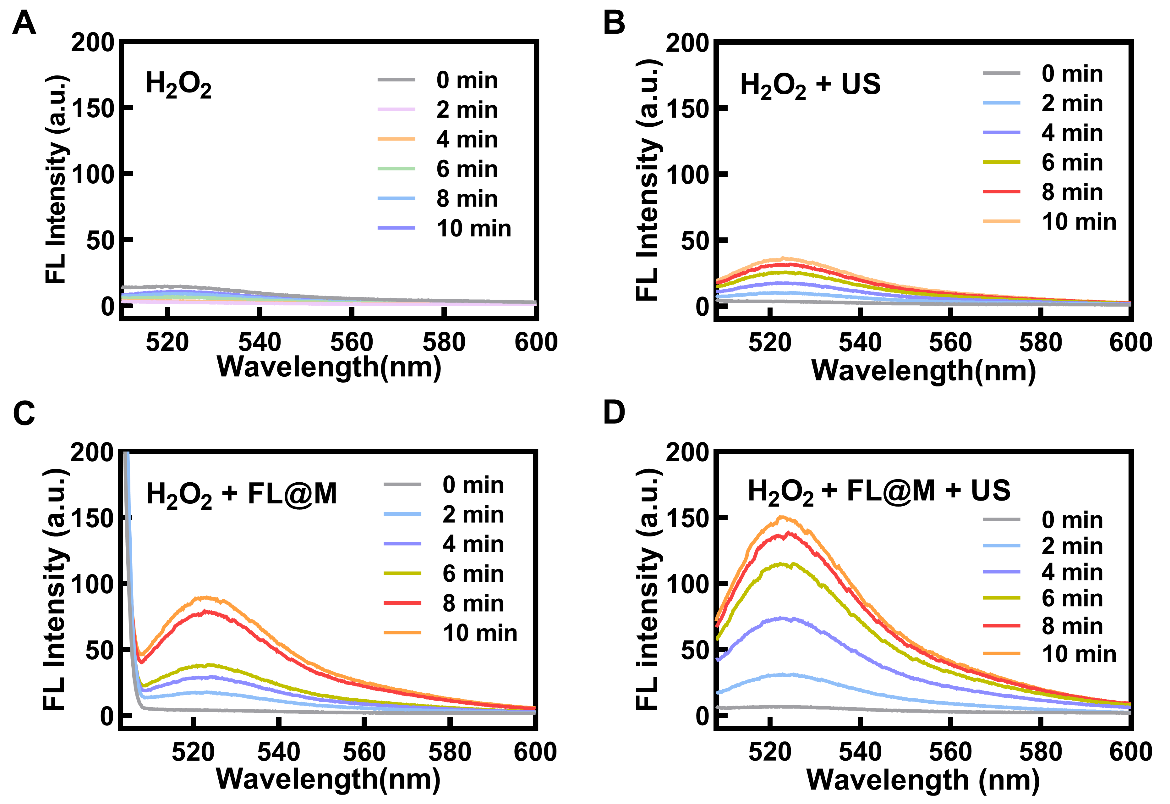


**Figure S4. Total ROS generation of FL@M after different treatments under US irradiation (1.0 W/cm^2^, 10 min) based on DCFH-DA assay.**


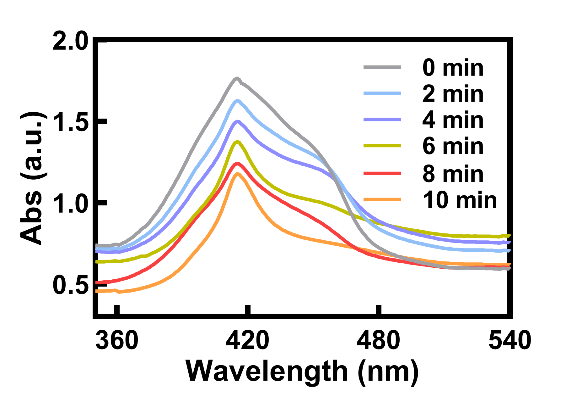


**Figure S5. The UV–vis absorption spectra of H_2_O_2_ + FL@M + US were detected by DPBF.**


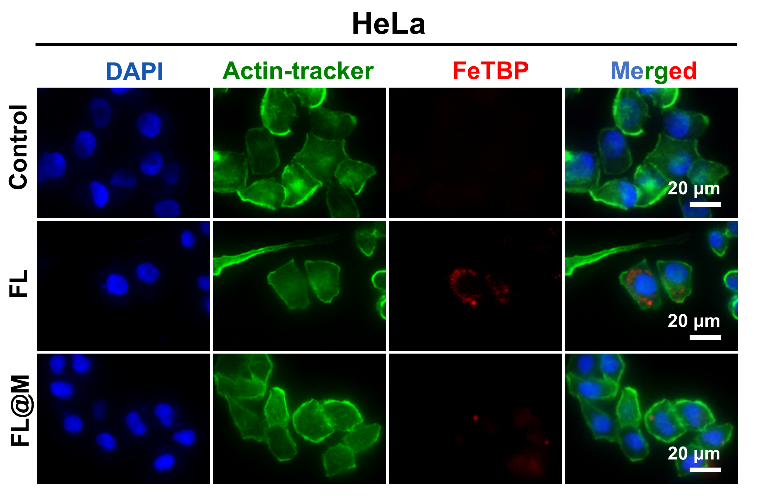


**Figure S6. Representative fluorescence images of HeLa cells incubated with FL or FL@M (coated with 8505C cell membrane) at 4 h.**


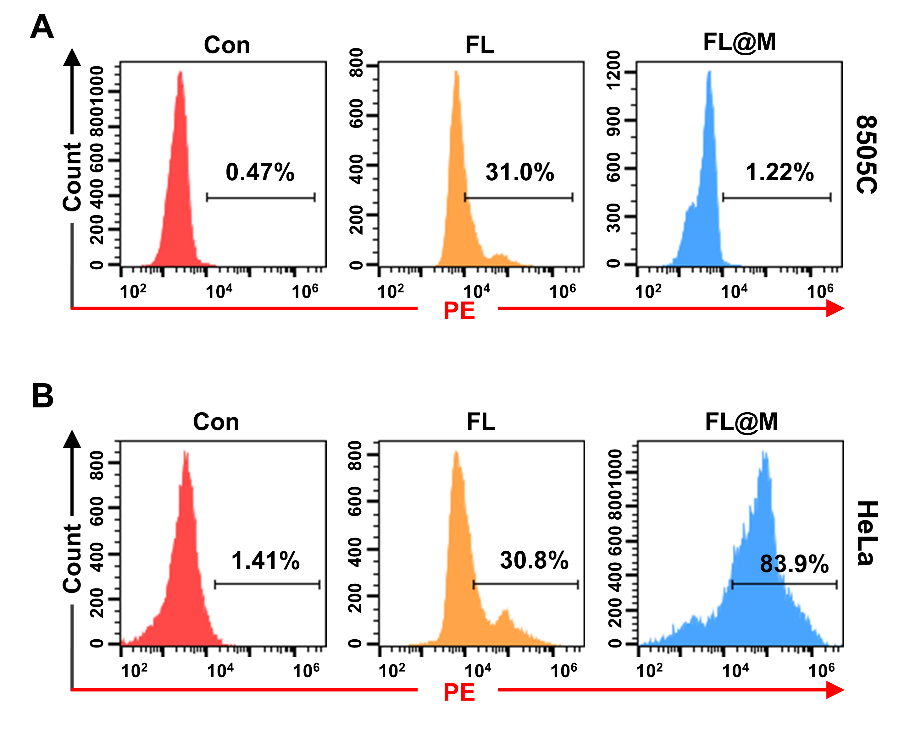


**Figure S7. Flow cytometry of 8505C cells (A) and HeLa cells (B) incubated with FL or FL@M (coated with HeLa cell membrane) at 4 h.**


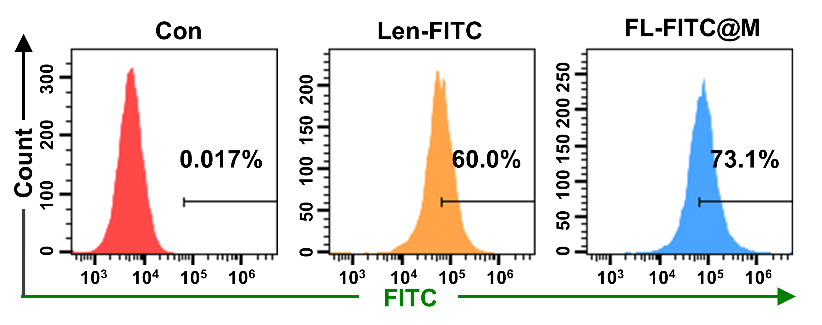


**Figure S8. Flow cytometry of 8505C cells incubated with Len-FITC or FL-FITC@M at 4 h.**


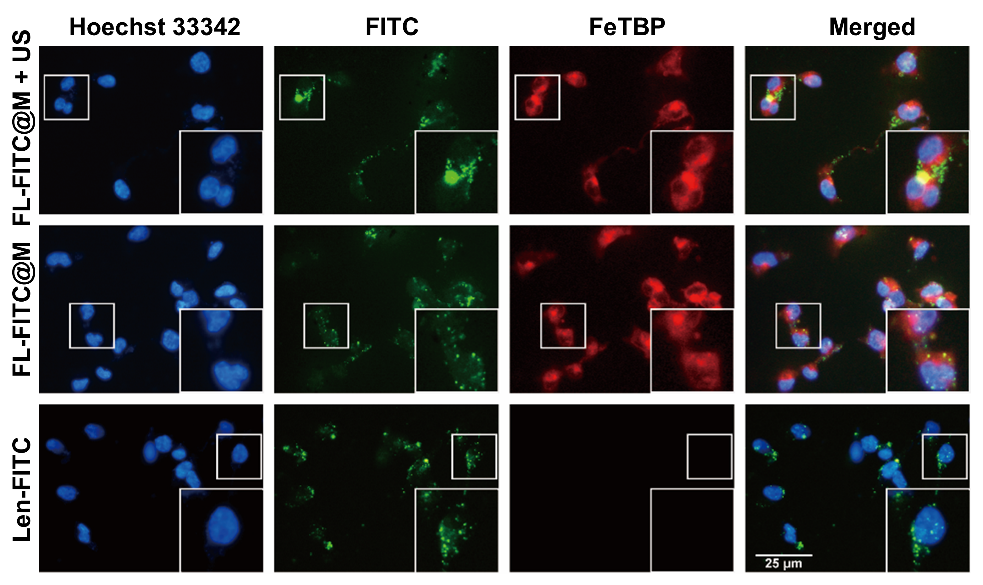


**Figure S9. Representative fluorescence images of 8505C cells incubated with Len-FITC or FL-FITC@M at 4 h.**


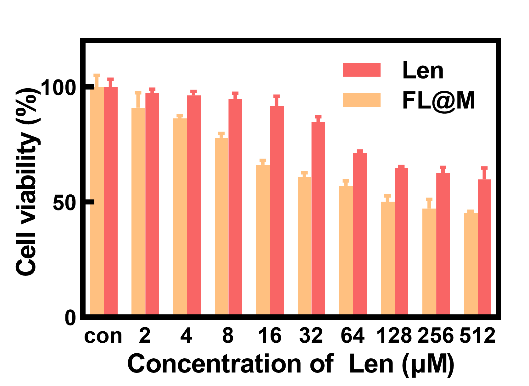


**Figure S10. Cell viabilities of 8505C treatment with Lenvatinib or FL@M by MTT (n = 3).**


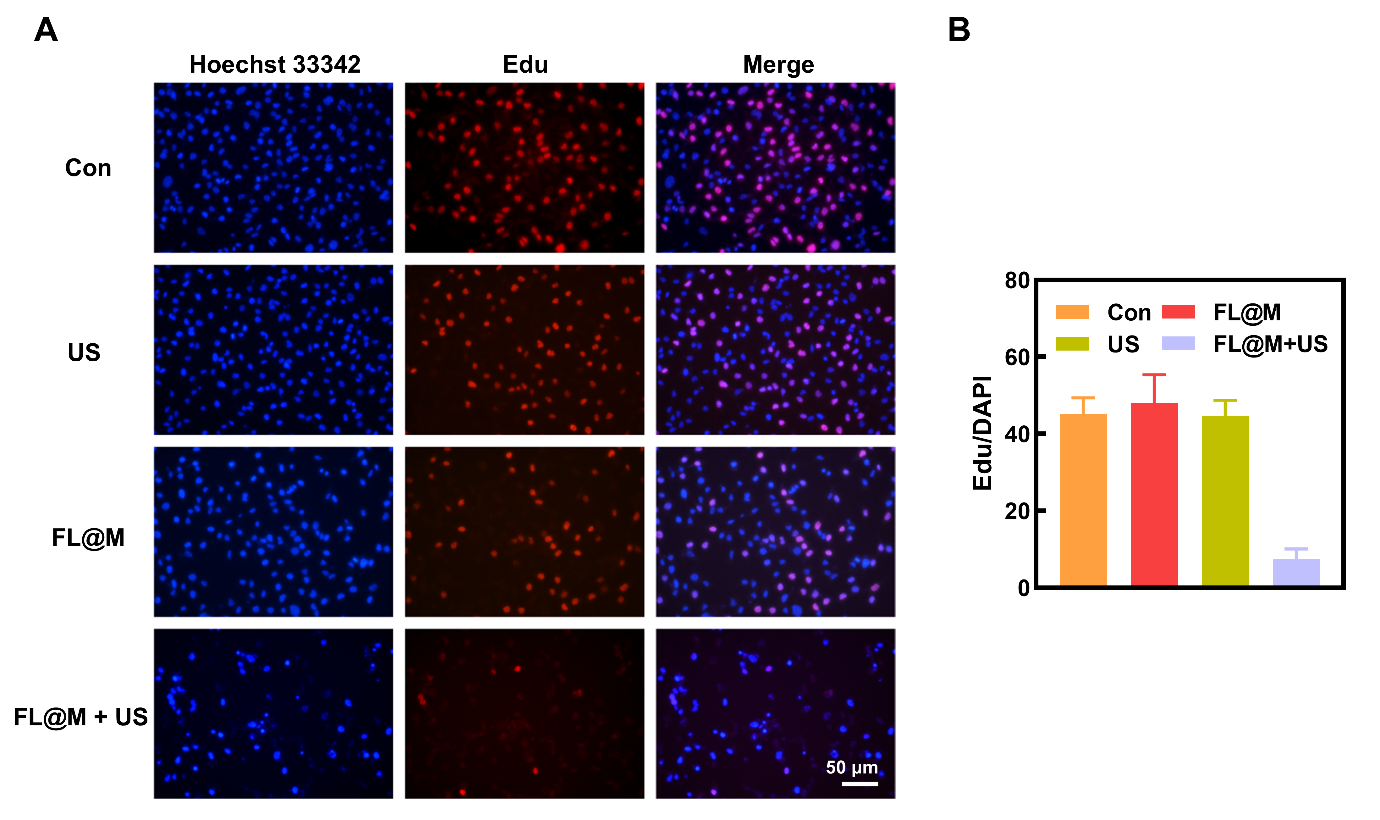


**Figure S11. Representative fluorescence images of Edu assays in 8505C cells after different treatments.**


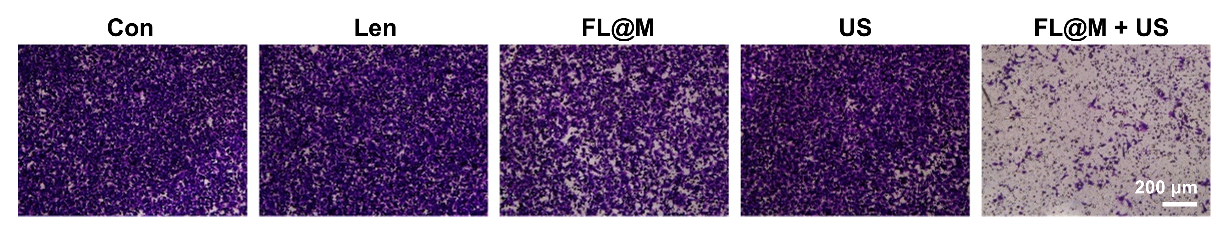


**Figure S12. Representative photos of** **transwell migration assays in 8505C cells after different treatments.**


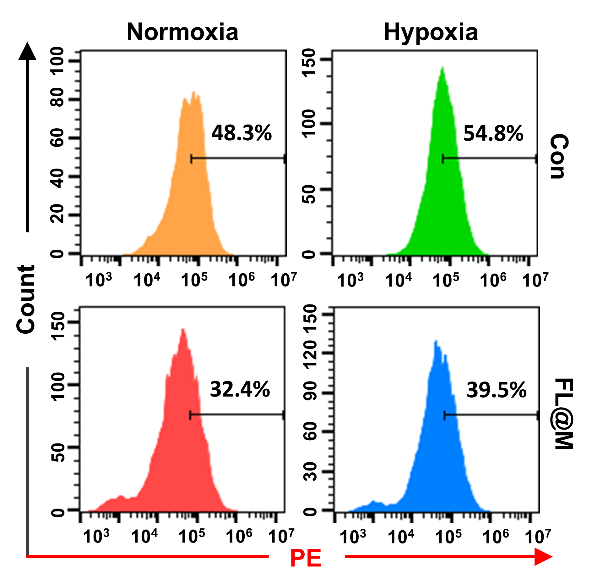


**Figure S13. Flow cytometry of intracellular O_2_ release detected by hypoxia probe (RDPP) under normoxia and hypoxia.**


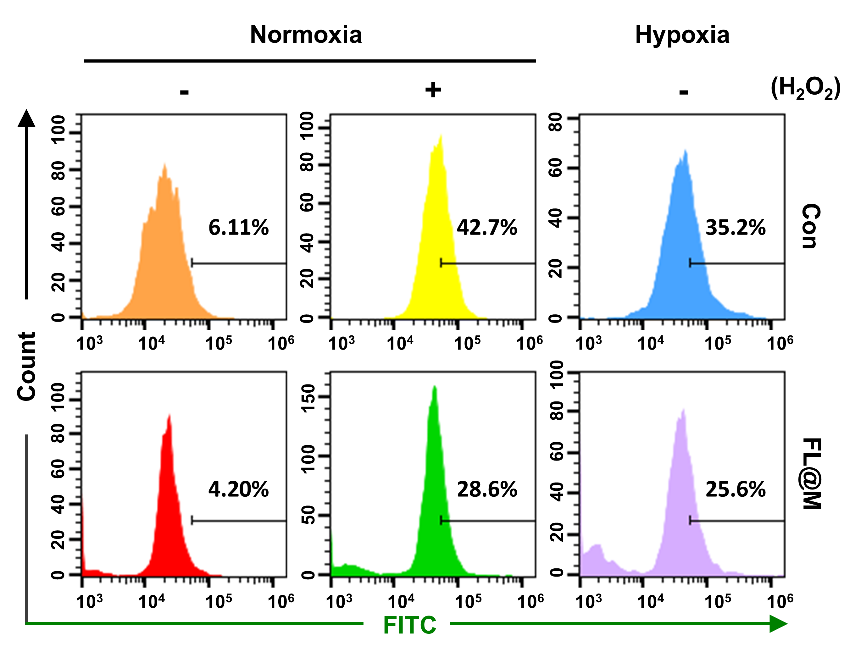


**Figure S14. Flow cytometry of intracellular H_2_O_2_ depleting detected by ROS Green™ Probe under normoxia and hypoxia.**


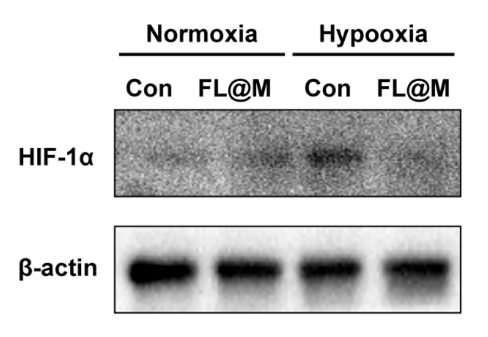


**Figure S15. Western blotting analysis of HIF-1α protein in 8505C cells with various treatments under normoxia and hypoxia.**

**
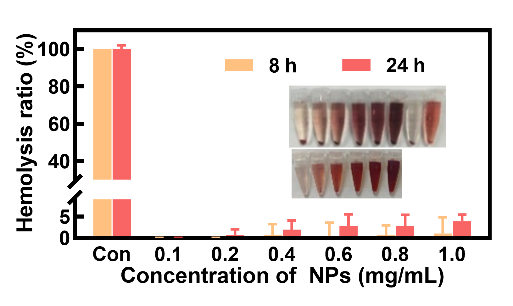
**

**Figure S16. Hemolysis after incubation with different concentrations of FL@M for 8 h and 24 h (n = 3).**

**
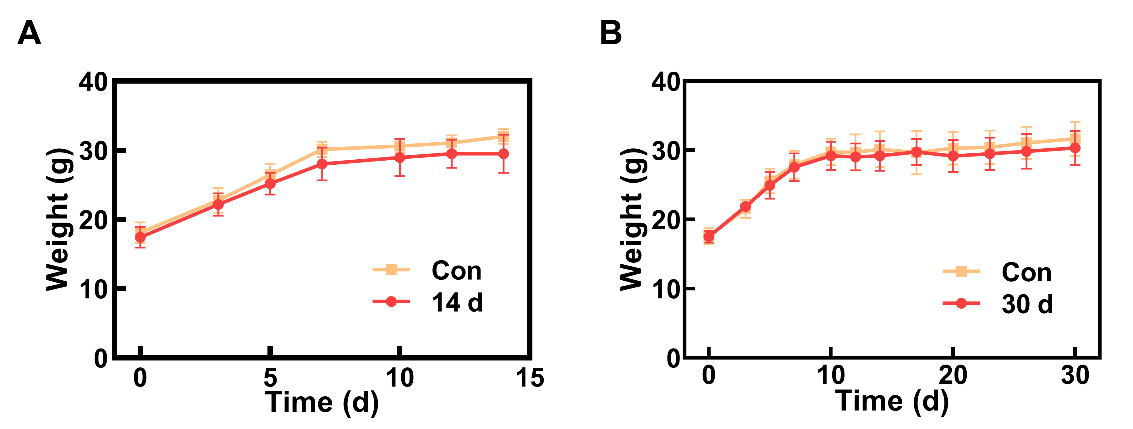
**

**Figure S17. Change in body weight of mice after injections FL@M for 14 d and 30 d (n = 3).**


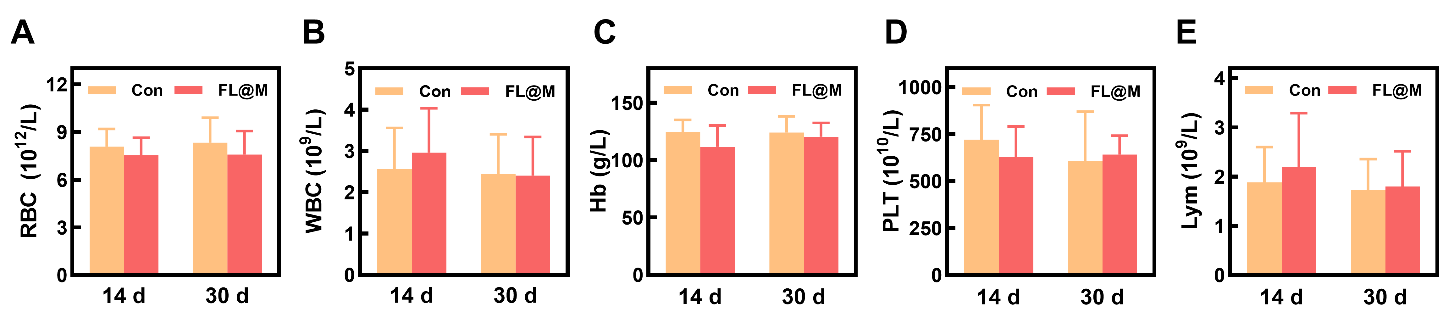


**Figure S18. The results of RBC, WBC, Hb, PLT, and Lym in different groups of mice (n = 3).**


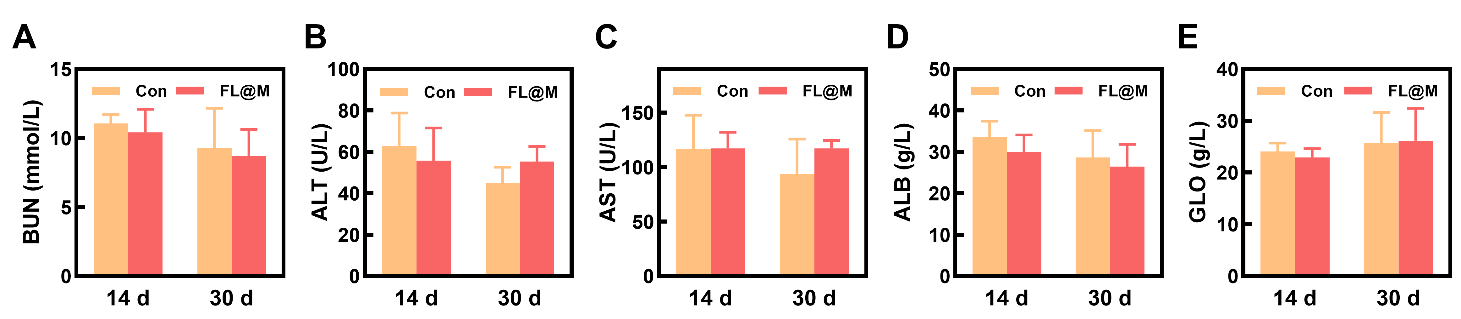


**Figure S19. The results of BUN, ALT, AST, ALB, and GLO in different groups of mice (n = 3).**


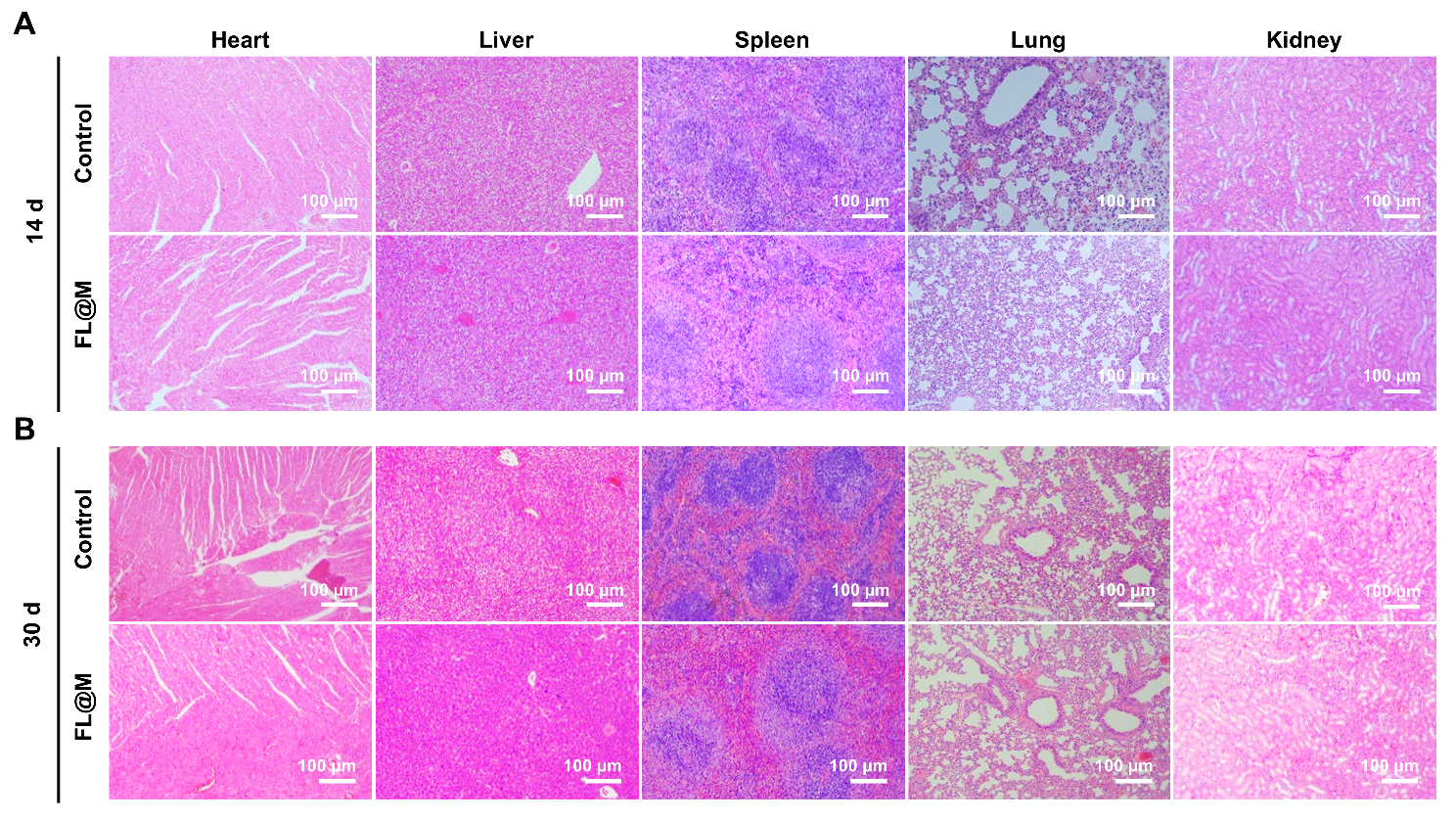


**Figure S20. H&E staining of major organs in mice after injections FL@M for 14 d (A) and 30 d (B).**

**Table S1.** The quantitative data of the Fe content of FeTBP by ICP-MS.


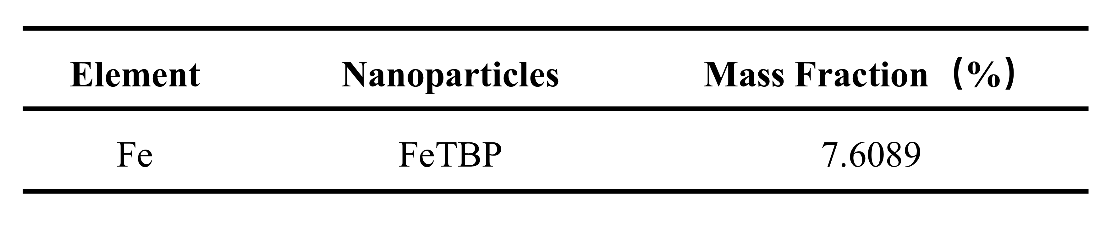

Supplement: Supplementary 1 — Figs. S1 to S20 Table S1 [file bmr.0338.f1.docx]
